# Fast Statistical Alignment

**DOI:** 10.1371/journal.pcbi.1000392

**Published:** 2009-05-29

**Authors:** Robert K. Bradley, Adam Roberts, Michael Smoot, Sudeep Juvekar, Jaeyoung Do, Colin Dewey, Ian Holmes, Lior Pachter

**Affiliations:** 1 Department of Mathematics, University of California Berkeley, Berkeley, California, United States of America; 2 Department of Molecular & Cellular Biology, University of California Berkeley, Berkeley, California, United States of America; 3 Department of Electrical Engineering & Computer Science, University of California Berkeley, Berkeley, California, United States of America; 4 Department of Bioengineering, University of California San Diego, San Diego, California, United States of America; 5 Department of Computer Sciences, University of Wisconsin, Madison, Wisconsin, United States of America; 6 Department of Biostatistics & Medical Informatics, University of Wisconsin, Madison, Wisconsin, United States of America; 7 Department of Bioengineering, University of California Berkeley, Berkeley, California, United States of America; Cornell University, United States of America

## Abstract

We describe a new program for the alignment of multiple biological sequences that is both statistically motivated and fast enough for problem sizes that arise in practice. Our **F**ast **S**tatistical **A**lignment program is based on pair hidden Markov models which approximate an insertion/deletion process on a tree and uses a sequence annealing algorithm to combine the posterior probabilities estimated from these models into a multiple alignment. FSA uses its explicit statistical model to produce multiple alignments which are accompanied by estimates of the alignment accuracy and uncertainty for every column and character of the alignment—previously available only with alignment programs which use computationally-expensive Markov Chain Monte Carlo approaches—yet can align thousands of long sequences. Moreover, FSA utilizes an unsupervised query-specific learning procedure for parameter estimation which leads to improved accuracy on benchmark reference alignments in comparison to existing programs. The centroid alignment approach taken by FSA, in combination with its learning procedure, drastically reduces the amount of false-positive alignment on biological data in comparison to that given by other methods. The FSA program and a companion visualization tool for exploring uncertainty in alignments can be used via a web interface at http://orangutan.math.berkeley.edu/fsa/, and the source code is available at http://fsa.sourceforge.net/.

## Author Summary

Biological sequence alignment is one of the fundamental problems in comparative genomics, yet it remains unsolved. Over sixty sequence alignment programs are listed on Wikipedia, and many new programs are published every year. However, many popular programs suffer from pathologies such as aligning unrelated sequences and producing discordant alignments in protein (amino acid) and codon (nucleotide) space, casting doubt on the accuracy of the inferred alignments. Inaccurate alignments can introduce large and unknown systematic biases into downstream analyses such as phylogenetic tree reconstruction and substitution rate estimation. We describe a new program for multiple sequence alignment which can align protein, RNA and DNA sequence and improves on the accuracy of existing approaches on benchmarks of protein and RNA structural alignments and simulated mammalian and fly genomic alignments. Our approach, which seeks to find the alignment which is closest to the truth under our statistical model, leaves unrelated sequences largely unaligned and produces concordant alignments in protein and codon space. It is fast enough for difficult problems such as aligning orthologous genomic regions or aligning hundreds or thousands of proteins. It furthermore has a companion GUI for visualizing the estimated alignment reliability.

## Introduction

The field of biological sequence alignment is very active, with numerous new alignment programs developed every year in response to increasing demand driven by rapidly-dropping sequencing costs. The list of approximately 60 sequence alignment programs on the wikipedia compilation provides a lower bound on the number of available tools and illustrates the confusing choice facing biologists who seek to select the “best” program for their studies. Nevertheless, the ClustalW program [1],[2], published in 1994, remains the most widely-used multiple sequence alignment program. Indeed, in a recent review of multiple sequence alignment [3], the authors remark that “to the best of our knowledge, no significant improvements have been made to the [ClustalW] algorithm since 1994 and several modern methods achieve better performance in accuracy, speed, or both.” Therefore, it is natural to ask, “Why do alignment programs continue to be developed, and why are new tools not more widely adopted by biologists?”.

A key issue in understanding the popularity of ClustalW is to recognize that it is difficult to benchmark alignment programs. Alignments represent homology relationships among the nucleotides, or amino acids, of the genomes of extant species, and it is impossible to infer the evolutionary history of genomes with absolute certainty. Comparisons of alignment programs therefore rely on databases of structural alignments for proteins and RNA or on gene loci or simulated data for DNA. Each type of benchmark is vulnerable to manipulation and furthermore may not represent the problem setups which are most relevant to biologists. The result is that biologists are confronted with many programs and publications, but it is frequently unclear which approach will give the best results for the everyday problems which they seek to address.

Adding to the difficulty of selecting software tools is the blur between programs and ideas. Developers of alignment programs make choices about the objective functions to optimize, the statistical models to use, and the parameters for these models, but the relative impact of individual choices is rarely tested [4]. Discordance among programs is frequently noted [5], but the different architectures of individual programs, and in some cases the lack of open software, makes it difficult to explore novel combinations of existing ideas for improving alignments.

In lieu of these issues, biologists have favored the conservative approach of using the tried and trusted ClustalW program, although they frequently use it in conjunction with software which allows for manual editing of alignments [6]. The rationale behind alignment-editing software is that trained experts should be able to correct alignments by visual inspection and that effort is better expended on manually correcting alignments than searching for software that is unlikely to find the “correct” alignment anyway. Although manual editing approaches may be cumbersome, they have been used for large alignments (e.g., [7]).

We therefore approached the alignment problem with the following goals in mind:

1. An approach which seeks to maximize the expected alignment accuracy. Our approach seeks to find the alignment with minimal expected distance to the true alignment of the input sequences, where the true alignment is treated as a random variable, with the probability of each true alignment determined under a statistical model. Explicitly incorporating a statistically-motivated objective function, this “expected accuracy” approach to alignment allows us to visualize alignments according to estimates of different quality measures, including their expected accuracy, sensitivity, specificity, consistency and certainty. We therefore offer biologists a way to compare alignments that allows them to quantitatively judge differences in alignment quality when they are performing manual edits.
2. A method which is robust to variation in evolutionary parameters. We sought a method which is robust to phenomena such as sequences of differing evolutionary distances and base composition. While “phylogenetic alignment” methods seek to accomplish this by explicitly modeling alignments on trees [8]–[11], a computationally-costly procedure, we use only pairwise comparisons of sequences and allow the pairwise model to vary for each pair considered.
3. Robust results when faced with the wide range of alignment problems encountered today. We sought to create a single program which is capable of achieving high accuracies on protein, RNA and DNA sequences without additional input from, e.g., database homology searches. We additionally sought to make our approach fast enough for large-scale problems such as aligning many sequences or orthologous regions of genomes. (When aligning genomic-size sequences, we assume that the sequences are collinear; we do not attempt to solve the problem of resolving duplications or inversions.)
4. Creation of a modular code base so that future improvements in one aspect of alignment could easily be incorporated into our approach. In particular, we aimed to create a collaborative infrastructure so that “bioinformaticians with expertise in developing software for comparing genomic DNA sequences [can] pool their ideas and energy to produce a compact tool set that serves a number of needs of biomedical researchers” [12].

The “distance-based” approach to sequence alignment, proposed in [13] and implemented in the protein alignment program AMAP [14], offers a useful framework for these goals. Much as distance-based phylogenetic reconstruction methods like Neighbor-Joining build a phylogeny using only pairwise divergence estimates, a distance-based approach to alignment builds a multiple alignment using only pairwise estimations of homology. This is made possible by the sequence annealing technique [14] for constructing multiple alignments from pairwise comparisons.

We have implemented our approach in FSA, a new alignment program described below. We give an overview of the structure of FSA and explain the details of its components below. Text S1 contains detailed instructions for using the FSA program and webserver as well as FSA's companion programs for comparing alignments and working with whole-genome alignments.

## Methods

### Overview

**Figure 1:**
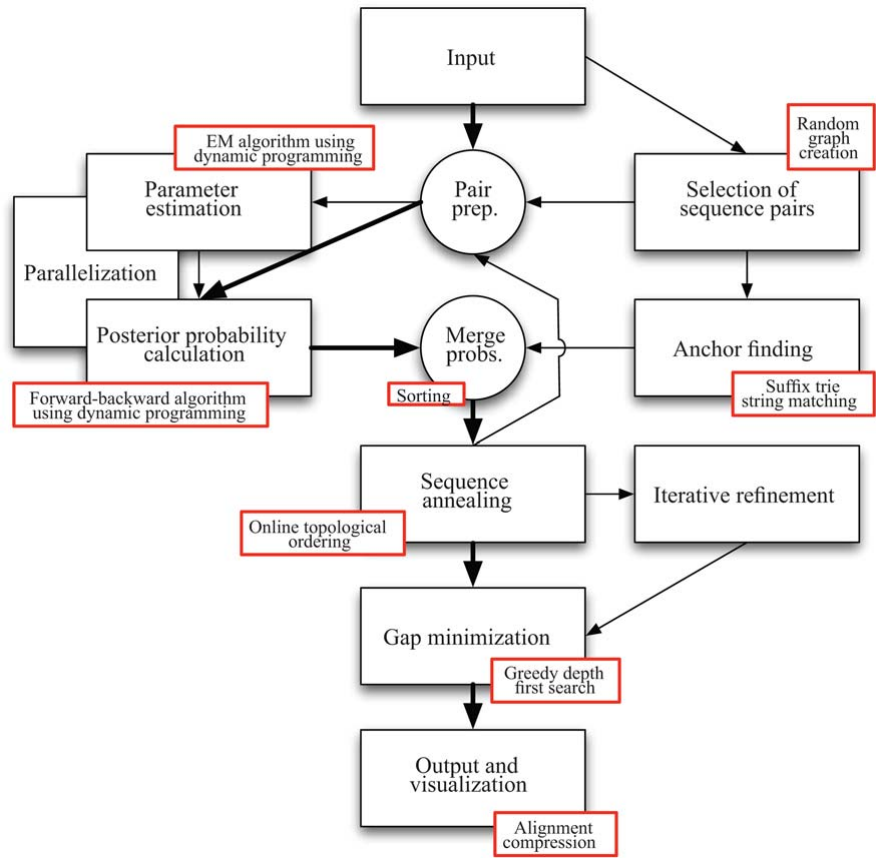
Overview of the components constituting the FSA alignment program. The algorithms that are used in each component are highlighted in the accompanying boxes. The bold arrows show the simplest mode of use for FSA, where posterior probabilities are calculated directly using default parameters for all pairs of sequences and the optional steps of anchor finding and iterative refinement are omitted.

Figure 1 shows an overview of the components of the FSA alignment algorithm, described in detail below.

The **input** to FSA is a set of protein, RNA or DNA sequences. These sequences are assumed to be homologous, although FSA is robust to non-homologous sequence. The **output** of FSA is a global alignment of the input sequences which is a (local) optima of the expected accuracy under FSA's statistical model.

FSA first performs pairwise comparisons of the input sequences to determine the posterior probabilities that individual characters are aligned (note, however, that it does not yet actually align any sequences). While the number of possible pairwise comparisons is quadratic in the number of sequences being aligned, giving unfavorable runtimes for datasets of many sequences, FSA overcomes this problem by reducing the number of pairs of sequences that are compared. This is accomplished using a randomized approach inspired by the Erdös-Rényi theory of random graphs, thereby drastically reducing the computational cost of multiple alignment.

After obtaining pairwise estimates of homology at the single-character level, FSA uses the sequence annealing technique [14] to construct a multiple alignment. This approach to alignment seeks to maximize the expected accuracy of the alignment using a steepest-ascent (greedy) algorithm.

The architecture of FSA reflects the inherent modularity of the distance-based approach to alignment. FSA's inference engine uses the flexible HMMoC code-generation tool [15] and has been parallelized with a separate module, alignments of long sequences are anchored with candidate matches found by the MUMmer suffix trie matching tool [16] or the exonerate homology-search program [17], and FSA's sequence annealing algorithm improves on the original algorithm and implementation in AMAP [14]. The stand-alone visualization tool uses statistical information produced by FSA, but otherwise is completely independent.

Each of these components can be improved independently of the others, allowing for rapid future improvements in distance-based alignment. For example, FSA's entire statistical model could easily be altered to incorporate position-specific features or completely replaced with a discriminative or hybrid generative-discriminative model.

### Core components

The components described here correspond roughly to the simplest mode of operation for FSA, outlined in bold in Figure 1.

#### Input and output

FSA accepts FASTA-format input files and produces alignments in multi-FASTA or Stockholm format. The server also outputs PHYLIP and ClustalW formatted files.

#### Estimating posterior probabilities of alignment

**Figure 2:**
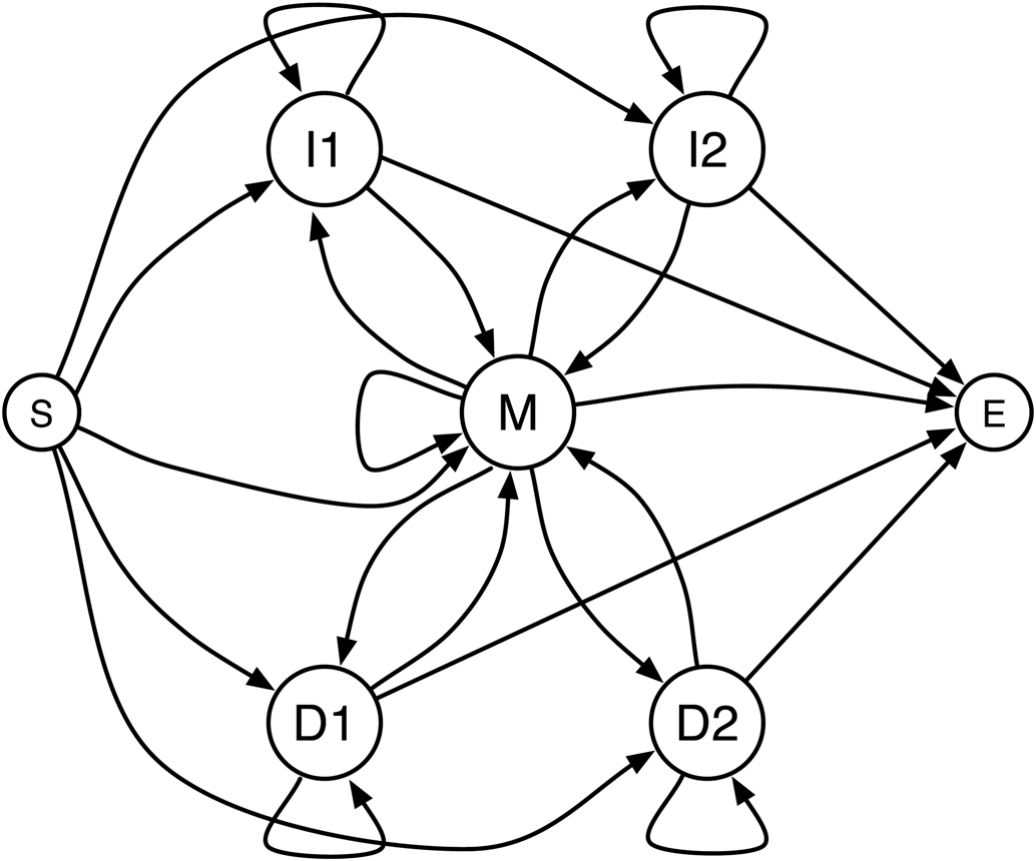
The default Pair HMM used by FSA. By default FSA uses a Pair HMM with two sets of Insert (I) and Delete (D) states to generate a two-component geometric mixture distribution. FSA can optionally use a three-state HMM, which has only one set of Insert and Delete states. M is a Match state emitting aligned characters.

Distance-based alignment, relying on pairwise estimations of homology, operates on the posterior probability distributions that characters in two sequences are aligned. FSA uses the standard three or five-state pair hidden Markov model (Pair HMM) shown in Figure 2 to infer these posterior probabilities of alignment, as well as posterior probabilities that characters are unaligned or gapped. The structure of the Pair HMM and its parameters can be controlled through the command-line options (see Text S1 for details).

The standard Forward-Backward algorithm on a Pair HMM has time complexity 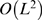 for two sequences of length 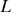.

#### Merging probabilities

After calculating the posterior probabilities of alignment for characters in all sequence pairs, 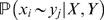 that individual characters 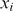 and 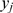 are aligned and 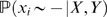 that a character 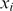 is gapped to sequence 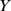, FSA sorts these probabilities according to a weighting function which gives a hill-climbing procedure which is a steepest-ascent algorithm in the weighting function (Text S1, “The mathematics of distance-based alignment”).

#### Sequence annealing

After estimating these posterior probabilities and sorting them, FSA creates a multiple alignment with the sequence annealing technique [14]. Sequence annealing attempts to find the alignment with the minimum expected distance to the truth (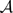), computed for two sequences 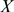 and 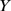 as
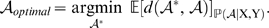



The distance 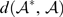 between two alignments is defined as the number of positions for which they make different homology statements, where the homology statement for 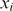 is either of the form 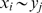 (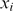 is homologous to 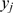) or 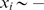 (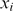 is not homologous to any position in 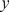) [14]. As a simple count of differing statements of homology (and non-homology), this distance has an intuitive biological interpretation. When one of the alignments is the true alignment, this distance can be thought of as the “evolutionary correctness” of the other, where the correctness of the alignment for each sequence position is equally weighted.

The alignment with the minimum expected distance to the truth is equivalent to the alignment with the maximum expected accuracy,
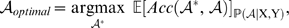
where we define the accuracy 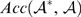 of an alignment 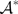 with respect to a reference, “true” alignment 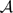 as the *fraction* of positions for which they make identical homology statements. In contrast with traditional measures of sensitivity and specificity, accuracy takes into account all positions, rather than just those that are predicted to have a homolog. (Note that it linearly penalizes incorrectly-placed gaps.)

The posterior probabilities over alignments 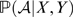 used in the optimization are given by FSA's statistical model (a Pair HMM). FSA extends this definition of an optimal pairwise alignment to an optimal multiple alignment by taking sum-of-pairs over all sequences.

Using this expected accuracy as an objective function for a greedy maximization, sequence annealing begins with the null alignment (all sequences unaligned) and merges single columns (aligns characters) according to the corresponding expected increase in 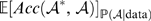, the similarity to the truth under FSA's statistical model. Whereas progressive alignment methods take large steps in alignment space by aligning entire sequences at each step, the distance-based approach takes the smallest-possible steps of aligning single characters.

“The mathematics of distance-based alignment” in Text S1 gives an in-depth discussion of the objective function and how it arises naturally from FSA's representation of an alignment as a partially ordered set (POSET) or directed acyclic graph (DAG).

#### Inferring indel events

**Figure 3:**
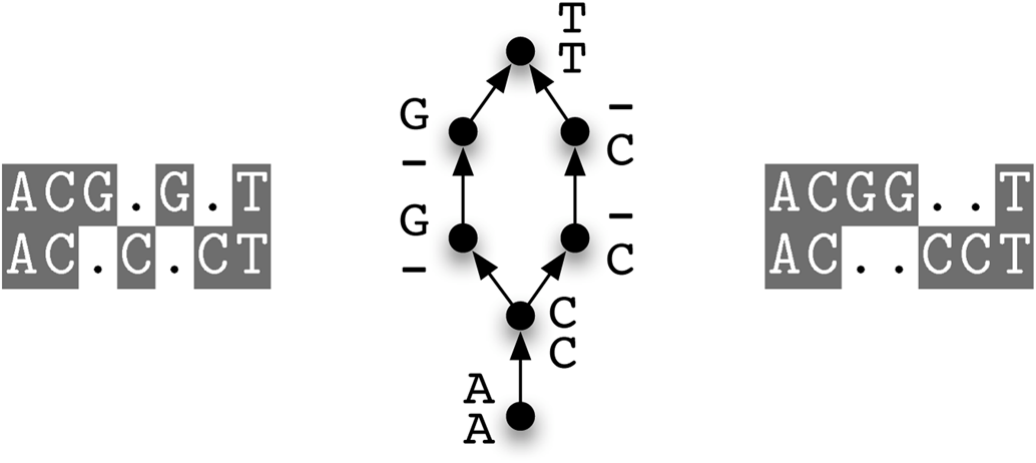
Two alignments (left and right) which make the same homology statements and therefore are both represented by the same POSET (center). “The mathematics of distance-based alignment” in Text S1 discusses this view of alignments as POSETs. The alignment on the right minimizes the number of gap-open events and as such is appropriate for analyses such as inferring parsimonious indel frequencies across a clade. Alignments are displayed with TeXshade [63].

In FSA's definition of an alignment, an alignment consists solely of a specification of homology. This definition differs from the standard definition of a multiple alignment, which implies an evolutionary history of substitution and indel events. For example, FSA (internally) considers the two alignments shown in Figure 3 to be equivalent.

This is problematic for comparative genomics analyses which use an alignment to infer evolutionary parameters such as indel frequencies across a clade. In order to output a single global alignment from which such evolutionary parameters can be inferred, we choose a topological ordering of the alignment POSET which corresponds to a maximum-parsimony interpretation of indel events. FSA outputs the global alignment with the minimum number of “gap openings” across the individual sequences (the right-hand alignment in Figure 3). As proved in Text S1, FSA can accomplish this in time linear in the number of sequences and sequence length.

### Optional components

#### Selection of a subset of pairs for alignment speedup

FSA can efficiently align hundreds or even thousands of sequences. By default it performs exhaustive distance-based alignment, which requires an all-pairs comparison of the 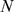 sequences, costing 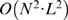. However, this prohibitive computational cost can be sharply reduced by only performing pairwise comparisons on a subset of all possible 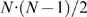 sequence pairs.

In order to ensure a complete alignment, where no sequence is left unaligned, each sequence must be connected to every other sequence by a series of pairwise comparisons. For 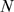 input sequences, a minimum of 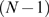 pairwise comparisons are necessary to give a complete alignment; this corresponds to building a spanning tree on the sequences. While this is sufficient to give a complete alignment on the input sequences, the results will depend heavily on which 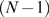 pairwise comparisons are used to construct the alignment and many choices may give poor alignments. Developing a good theory of which pairs to use to construct the best alignment with the fewest comparisons—how to select a randomized subset of pairs for comparison which falls between the extremes of 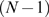 and 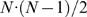 pairs—remains an open problem.

We therefore chose to use a completely-randomized approach inspired by results from the theory of Erdös-Rényi random graphs. By the Erdös-Rényi theory, a random graph will almost surely be connected if the edge-creation probability satisfies 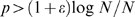, which is very low for large 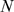 (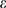 is a small positive number). Guided by this result, FSA performs fast alignments by first constructing a spanning tree on the input sequences and then performing each possible pairwise comparison with some probability 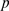 proportional to the connectedness threshold. The savings are dramatic—for 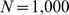 sequences, the Erdös-Rényi threshold probability is 0.007, corresponding to an algorithm which is over 100 times as fast as an all-pairs comparison—and we have observed little loss of accuracy from considering only a subset of pairs.

This speedup reduces the time complexity of both inference and sequence annealing. The cost of inference is reduced to 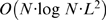 and the “worst average case” runtime of sequence annealing to 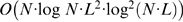, where we align 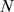 sequences of length 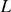 by making 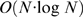 pairwise comparisons (Text S1, “The mathematics of distance-based alignment”).

#### Finding anchors

FSA can align megabase-long sequences using an “anchor annealing” strategy. Analogously to other genome alignment programs such as MAVID [18], MLAGAN [19], CHAOS/DIALIGN [20] and Pecan [21], FSA uses long matches to anchor regions of the alignment and performs inference with dynamic programming in between anchors. FSA's basic anchoring mode uses the fast suffix trie matching program MUMmer [16] to find candidate anchors and can find anchors in either nucleotide or protein space (by translating the sequence in all frames). FSA requires that anchors be maximal unique matches in both sequences (“MUMs”). The restriction to unique matches helps to prevent false-positive anchors due to, e.g., repetitive sequence; for example, a microsatellite can appear as a candidate anchor only if it appears exactly once, with identical copy number, in each sequence.

FSA utilizes its distance-based approach to find a consistent set of anchors across all sequences simultaneously, thereby making maximal use of additional constraints from other sequences. This “anchor annealing” strategy is conceptually similar to the procedures used in programs for aligning long sequences such as CHAOS/DIALIGN, MAVID, Pecan and TBA, which return partially-ordered sets of anchors, thereby permitting constraints to be projected across multiple sequences.

As with sequence annealing, this “anchor annealing” can be accomplished efficiently with a greedy algorithm based on the Pearce-Kelly algorithm. FSA uses the same code for both sequence and anchor annealing, although the objective function is different: Anchor “scores” correspond to *p*-values under a null model rather than probabilities of homology, and so there are no “gap” probabilities 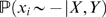 or 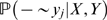 which contribute to the anchor-annealing analog of the expected accuracy 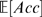.

Rather than aligning entire anchors across the multiple alignment in order to find a consistent set of anchors, FSA finds a set of anchor centroids which are consistent across all sequences and then prunes the resulting anchors at a pairwise level. The result is a set of anchors between pairs of sequences whose centroids are consistent across all sequences and which are non-overlapping between pairs of sequences. This heuristic approach permits FSA to quickly find consistent anchors across many sequences.

After finding a consistent set of anchors across the multiple alignment, FSA assumes that these anchors are correctly aligned with probability ∼1 and then aligns the regions between anchors using dynamic programming. When anchored alignment is performed, the posterior probabilities that individual characters are aligned, which FSA uses to inform construction of the multiple alignment, are conditioned on the set of anchors chosen. Therefore, if all anchors correspond to true homology then these probabilities will be correctly estimated despite the anchoring heuristic; however, if incorrect anchors are chosen, then individual probabilities of alignment can be similarly incorrect.

While FSA's restriction of candidate anchors from MUMmer to MUMs produces a very specific set of anchors, the restriction can be too stringent to obtain sensitive alignments of diverged or very long sequences, for which there are few unique exact matches. To address this potential problem, FSA can use the fast homology-search program exonerate [17] to find inexact matches to use as anchors as well. Furthermore, when performing whole-genome alignment, homologous genomic regions are typically first identified with a program such as Mercator [22] and then each region is aligned with a nucleotide-level alignment program. FSA can use the seed matches, frequently coding genes, which define the homologous genomic regions to inform its anchor annealing.

Because FSA uses the fast tool MUMmer to find anchors, it can rapidly align closely-related sequences, such as mitochondrial DNA, for which anchors span most of the alignment, making costly dynamic programming largely unnecessary.

#### The Pair HMM and parameter estimation

The distinct functional constraints acting on biological sequences give rise to very different patterns of molecular evolution, each implying distinct parameterizations of an appropriate model for alignment. For example, if substitutions or indels are more frequent in one lineage than in the others, then using a single model for all sequences (which does not reflect these differing constraints) can give misleading results. Nonetheless, sequence alignment algorithms traditionally use a single model for all sequences.

In order to overcome these difficulties, FSA uses “query-specific learning,” wherein a different model is learned for each pairwise comparison (the “query”). This is done in a completely unsupervised framework: FSA uses an unsupervised Expectation Maximization (EM) algorithm to estimate transition (gap) and emission (substitution) probabilities of the Pair HMM on the fly.

Despite its unsupervised nature, FSA's query-specific learning needs remarkably little sequence data to effectively learn parameters. Standard alignment algorithms estimate parameters from thousands or tens of thousands of pairs of *aligned* sequences; in contrast, we empirically observe good results with as little input data as two *unaligned* DNA or RNA sequences of length 60 nucleotides or four *unaligned* protein sequences of length 266 amino acids. These figures correspond to observing each of the independent parameters of a substitution matrix four times.

While FSA learns distinct transition parameters for every pair of query sequences regardless of the sequence composition, it uses different learning strategies for nucleotide and amino acid emission matrices. Because a pair emission matrix over aligned nucleotides has only (4^2^−1) = 15 free parameters, FSA can learn a different emission distribution for every pairwise comparison of all but the shortest RNAs or DNAs (this allows FSA to learn a different model for each pair of unanchored subsequences when performing anchored aligment). In contrast, emission matrices over aligned amino acids have (20^2^−1) = 3,999 free parameters, thereby preventing FSA from learning independent models for each pair of proteins. FSA therefore learns a single emission matrix using an all-pairs comparison for protein sequences.

Because FSA uses unsupervised learning on very sparse data, overfitting is a serious concern. FSA attempts to prevent overfitting by (1) using a weak Dirichlet regularizer (prior) when estimating both transition and emission probabilities, and (2) terminating parameter learning before the EM algorithm converges. By default the Dirichlet emission priors are scaled such that total number of pseudocounts for aligned characters is equal to the number of free parameters in a symmetric pair emission matrix. As is the case for other machine-learning algorithms, it can be shown that termination before convergence of query-specific learning is equivalent to a form of regularization (likelihood penalty).

If there is insufficient sequence data for effective learning, then FSA does not estimate parameters and instead uses default parameters to construct an alignment. The default parameters values, as well as seeds used for the learning algorithm, are discussed in Text S1.

#### Parallelization mode

While FSA can align hundreds or thousands of sequences on a single computer, it can handle truly large-scale problems by running in a parallelized mode on a computer cluster. FSA's distance-based approach to alignment gives the multiple alignment a natural independence structure: each pairwise alignment is independent of all other pairs, allowing dramatic runtime reduction by distributing the individual pairwise computations to different processors.

Two factors were considered for the parallelization of FSA : (1) communication overhead between nodes, and (2) workload distribution over the different processors. For example, distributing jobs in very small batches may reduce processor idle time but lead to high overhead; in contrast, using large batches may increase idle time but minimize overhead. FSA's parallelization mode uses the “Fixed-Size Chunking” strategy described in [23], whereby each of the 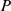 processors runs on chunks of 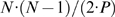 pairwise comparisons.

**Figure 4:**
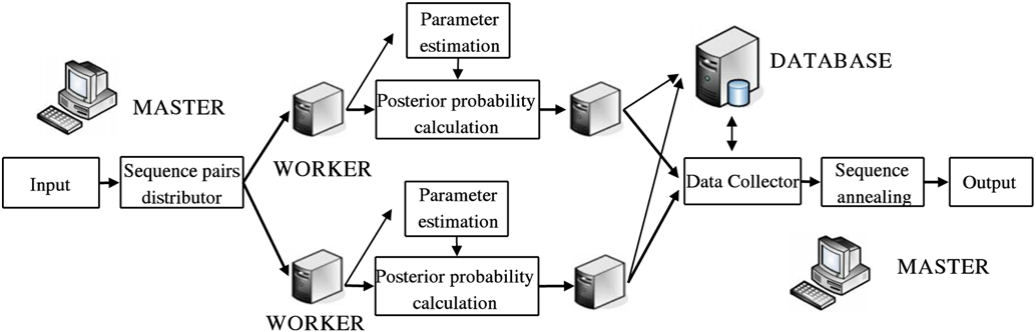
Schematic overview of FSA's parallelization strategy on a computer cluster. For large input sizes, a disk-based database may be used to store some of the primary data structures and reduce memory usage.

While the pairwise comparisons can be naturally parallelized, sequence annealing does not have the same obvious independencies. Therefore, even when running in parallelized mode, FSA performs sequence annealing on a single node. The parallelization of the annealing step is a future aim for this project. A schematic of the current parallelization strategy is given in Figure 4.

#### Iterative refinement

As a greedy algorithm, sequence annealing is only guaranteed to find a local optima of the expected accuracy. FSA therefore uses an iterative refinement strategy after sequence annealing terminates to locally improve the alignment. For each round of iterative refinement, FSA looks at every character's position in the multiple alignment and sees whether the objective function can be improved by moving it to another position (without violating the consistency constraints of the multiple alignment). FSA assembles a list of such candidate character shifts, orders the list by the expected change in the objective function, and then performs the shifts. Iterative refinement terminates when there are no candidate shifts which improve the objective function.

#### Visualization

**Figure 5:**
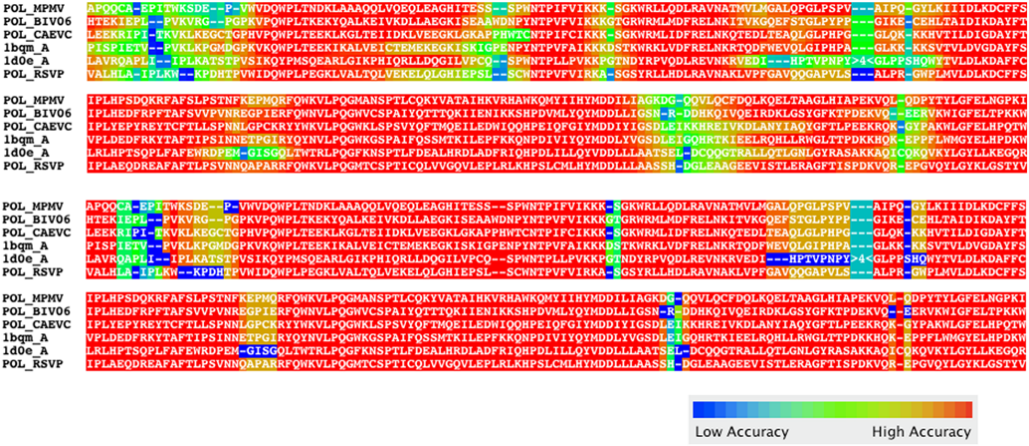
The Java GUI allows users to visualize the estimated alignment accuracy under FSA's statistical model. FSA's alignment is colored according the expected accuracy under FSA's statistical model (top) as well as according to the “true” accuracy (bottom) given from a comparison between FSA's alignment and the reference structural alignment. It is clear from inspection that accuracies estimated under FSA's statistical model correspond closely to the true accuracies. Sequences are from alignment BBS12030 in the RV12 dataset of BAliBASE 3 [24].

FSA's included GUI permits the user to visually assess alignment quality under FSA's statistical model according to estimates of different measures, including expected accuracy, sensitivity, specificity, consistency and certainty. This permits biologists and bioinformaticians to incorporate reliability measures into downstream analyses, such as evolutionary rate measurements and phylogenetic reconstruction. Incorporating such information can produce distinctly different results. For example, over-aligned non-conserved sequence can cause a systematic bias towards long branch lengths; this can be ameliorated by incorporating the expected accuracy statistics produced by FSA into reconstruction algorithms. Figure 5 shows a sample protein alignment colored by the expected alignment accuracy under FSA's statistical model as well as the true accuracy (based on a reference structural alignment).

FSA's GUI can color alignments according to five different measures of alignment quality, which are approximated under its statistical model. Characters 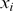 in a multiple alignment can be colored according to:

- Accuracy: The expected accuracy with which 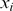 is aligned to other characters or gaps.
- Sensitivity: The expected sensitivity with which 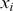 is aligned to other characters.
- Specificity: The expected specificity with which 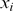 is aligned to other characters.
- Certainty: The certainty with which 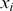 is aligned correctly (whether there is a good alternate choice).
- Consistency: The consistency of the many pairwise comparisons used to construct the multiple alignment and the implied optimality of the alignment of 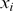 to other characters or gaps in the multiple alignment.

See Text S1 for detailed descriptions of how these measures are defined and calculated using FSA's statistical model.

The GUI also provides a visual and statistical guide when manually editing alignments.

## Results

We benchmarked FSA against databases of multiple alignments compiled from reference structural alignments, including protein databases (BAliBASE 3 [24] and SABmark 1.65 [25]), small RNA databases (BRAliBase 2.1 [26]), large RNA databases (Consan mix80 [27]), and both mammalian [28] and fly [29],[30] simulated DNA alignments.

Alignment programs are commonly used to detect homology among input sequences. We conducted a series of false-positive experiments to test whether commonly-used alignment programs can reliably identify homologous and non-homologous sequence. Surprisingly, we found that for most alignment programs, aligned sequences are not necessarily homologous, indicating that biologists should use caution when interpreting the multiple alignments produced by many commonly-used tools.

We additionally performed a simple test of the consistency of common programs when aligning coding sequence: We aligned 1,502 genes orthologous across seven species of yeast in both nucleotide and protein space and compared the resulting alignments. Many programs gave surprisingly discordant results, indicating that at least one of these two alignments produced by commonly-used programs is incorrect.

### Protein sequence

Table 1 shows benchmarks of FSA and other alignment programs, including AMAP [14], ClustalW [1],[2], DIALIGN [31],[32], MAFFT [33], MUMMALS [34], MUSCLE [35], Probalign [36], ProbCons [37], T-Coffee [38], and SeqAn::T-Coffee [39], against the BAliBASE 3 [24] and SABmark 1.65 databases [25]. FSA in maximum-sensitivity mode had accuracy similar to those of the better-performing programs on BAliBASE 3 and had accuracy superior to that of any other program on SABmark 1.65 when run in default mode. FSA had higher positive predictive values than any other tested program on all datasets. Remarkably, FSA was the only tested program which achieved a mis-alignment rate <50% on the standard SABmark 1.65 datasets; all other programs produced more incorrect than correct homology statements.

**Table 1:** Benchmarks against protein structural databases.

Program	BAliBASE 3	BAliBASE 3+fp	SABmark 1.65
	(Acc/Sn/PPV)	(Acc/Sn/PPV)	(Acc/Sn/PPV)
AMAP	0.70/0.62/0.83	0.73/0.61/0.80	0.57/0.43/0.46
ClustalW	0.66/0.63/0.62	0.59/0.63/0.53	0.38/0.44/0.30
DIALIGN	0.68/0.63/0.68	0.68/0.62/0.63	0.48/0.41/0.34
FSA	0.71/0.62/0.85	**0.75**/0.62/0.84	**0.59**/0.38/0.52
FSA (–maxsn)	0.73/0.68/0.76	0.74/0.68/0.72	0.52/0.45/0.39
MAFFT	0.74/0.71/0.71	0.68/0.71/0.61	0.44/0.49/0.35
MUMMALS	0.74/0.70/0.73	0.69/0.70/0.64	0.49/0.52/0.38
MUSCLE	0.70/0.67/0.66	0.63/0.66/0.57	0.40/0.46/0.32
Probalign	**0.76**/0.72/0.73	0.71/0.71/0.65	0.49/0.50/0.37
ProbCons	0.74/0.70/0.72	0.69/0.70/0.64	0.47/0.50/0.37
T-Coffee	0.72/0.67/0.71	0.67/0.67/0.63	0.45/0.46/0.35
SeqAn::T-Coffee	0.73/0.69/0.70	0.67/0.69/0.61	0.43/0.47/0.34

Comparisons of the accuracies (Acc), sensitivities (Sn) and positive predictive values (PPV) of FSA and other alignment methods on the BAliBASE 3 [24] and SABmark 1.65 [25] databases. Probalign has the highest accuracy on the commonly-used BAliBASE 3 dataset and FSA in default mode has superior accuracy on the BAliBASE 3+fp and SABmark 1.65 datasets (note that only FSA and AMAP explicitly attempt to maximize the expected accuracy). FSA has higher positive predictive values than any other program on all datasets and can additionally achieve high sensitivity when run in maximum-sensitivity mode. The BAliBASE 3+fp dataset, which mirrors BAliBASE 3 but includes a single non-homologous sequence in each alignment, was designed to test the robustness of alignment programs to incomplete homology. Traditional alignment programs, designed to maximize sensitivity, suffer greatly-increased mis-alignment when even a single non-homologous sequence is introduced; in contrast, FSA is robust to the non-homologous sequence and has an unchanged positive predictive value. Remarkably, FSA was the only tested program with a mis-alignment rate of <50% on the SABmark 1.65 dataset; the majority of the homology statements made by other programs were incorrect. Because the SABmark 1.65 dataset contains many sequences of only partial or even no homology, a method such as FSA which is robust to non-homologous sequence performs better under our accuracy criterion than a program such as MUMMALS despite the fact that MUMMALS has significantly-higher sensitivity on this dataset. The BAliBASE 3 dataset consisted of full-length sequences in all reference sets RV11, RV12, RV20, RV30, RV40 and RV50; we created the BAliBASE 3+fp dataset from the same reference sets by adding a single false-positive, a random sequence, to each alignment. The SABmark 1.65 dataset consisted of the Twilight Zone and Superfamilies datasets.

In order to test the robustness of alignment programs to incomplete homology, we modified the BAliBASE 3 database such that every alignment included a single false-positive, an unrelated (random) sequence. This is a realistic setup for biologists who might want to decide whether a sequence is orthologous to a particular protein family. With the exception of FSA, the tested alignment programs suffered a false-positive rate increased by over 25% on this BAliBASE 3+fp dataset, indicating that they aligned the random sequence with the homologous set. In contrast, FSA left the random sequence unaligned and had an essentially-unchanged false-positive rate.

### RNA sequence

Table 2 shows benchmarks of FSA and the other tested alignment programs against the BRAliBase 2.1 [26] and Consan mix80 [27] databases. FSA outperformed all other programs on both datasets.

**Table 2:** Benchmarks against RNA structural databases.

Program	BRAliBase 2.1	Consan mix80
	(Acc/Sn/PPV)	(Acc/Sn/PPV)
ClustalW	0.85/0.86/0.86	0.65/0.65/0.68
DIALIGN	0.82/0.83/0.85	0.76/0.75/0.82
FSA	0.90/0.91/0.94	0.77/0.74/0.92
FSA (–maxsn)	**0.91**/0.92/0.92	**0.78**/0.78/0.86
MAFFT	0.90/0.91/0.91	0.77/0.78/0.77
MUSCLE	0.90/0.91/0.90	0.74/0.76/0.74
ProbConsRNA	0.91/0.92/0.92	(failed to align)
T-Coffee	0.81/0.82/0.84	0.38/0.33/0.40
SeqAn::T-Coffee	0.89/0.90/0.90	(failed to align)

Comparisons of the accuracies (Acc), sensitivities (Sn) and positive predictive values (PPV) of FSA and other alignment methods on the BRAliBase 2.1 dataset of small RNAs [26] and the Consan mix80 dataset of Small and Large Subunit ribosomal RNAs [27]. The BRAliBase 2.1 dataset consisted of all alignments with 15 sequences (the largest alignments). The mix80 dataset provided difficult alignment problems: The four alignments each contain from 107 to 254 sequences of approximately 1–4 kilobases in length, with average percentage identity less than <50%. Two program, ProbConsRNA and SeqAn::T-Coffee, were incapable of aligning these large datasets. When run in –fast mode, FSA considers only a subset (∼20% in this case) of all sequence pairs. Note that because the mix80 dataset consists of long sequences, FSA automatically uses anchoring for speed. FSA does not use anchoring on the short sequences of BRAliBase 2.1.

BRAliBase 2.1 was assembled from the RFAM [40] RNA database and consists of small RNAs (average length of ∼150 nt). FSA gave improved performance even on this high-identity dataset where most programs did relatively well.

The Consan mix80 dataset of alignments of Small and Large Subunit ribosomal RNAs, assembled from the European Ribosomal RNA database [41], was created for training RNA structural alignment programs and provides a test of alignment programs on difficult, large-scale alignments. The four alignments contain from 107 to 254 sequences, each 1–4 kilobases in length, with average percentage identity less than <50%. Two tested alignment programs, ProbConsRNA [42] and SeqAn::T-Coffee, were unable to align these large datasets. This dataset demonstrates that FSA's alignment speedup options, including performing inference only on a subset of all possible pairs (–fast) and anchoring alignments instead of using the full dynamic programming matrix (–anchored), are effective heuristics for large datasets.

### DNA sequence

Table 3 shows benchmarks of FSA and other genomic alignment programs, including CHAOS/DIALIGN [20], DIALIGN-TX [31],[32], MAVID [18], MLAGAN [19], Pecan [21] and TBA [28], on simulated alignments of both mammalian and *Drosophila* DNA sequences. FSA produced higher-accuracy alignments than the other programs on the two *Drosophila* datasets and only Pecan gave better alignments of the mammalian sequences.

**Table 3:** Benchmarks against simulated mammalian and fly genomic DNA.

Program	Blanchette et al.	DAWG	simgenome
	(Acc/Sn/PPV)	(Acc/Sn/PPV)	(Acc/Sn/PPV)
CHAOS/DIALIGN	0.58/0.44/0.74	0.72/0.46/0.43	0.62/0.67/0.59
DIALIGN-TX	0.73/0.68/0.77	0.72/0.51/0.44	0.64/0.68/0.61
FSA (–exonerate)	0.86/0.82/0.93	**0.81**/0.38/0.74	**0.79**/0.78/0.84
FSA (–exonerate –maxsn)	0.87/0.85/0.90	0.75/0.41/0.50	0.76/0.79/0.77
MAVID	0.57/0.45/0.68	0.66/0.36/0.32	0.72/0.77/0.72
MLAGAN	0.70/0.63/0.80	0.45/0.39/0.19	0.71/0.71/0.73
Pecan	**0.92**/0.91/0.92	0.77/0.48/0.53	0.78/0.81/0.78
TBA	0.83/0.81/0.87	0.80/0.32/0.75	0.74/0.79/0.72

Comparisons of the accuracies (Acc), sensitivities (Sn) and positive predictive values (PPV) of FSA and other alignment methods on simulated alignments of mammalian and *Drosophila* DNA. The simulated alignments of nonfunctional DNA sequences (“Blanchette et al.”) from nine mammals (human, chimp, baboon, mouse, rat, cat, dog, cow, and pig) were produced by [28]. Simulated alignments of nonfunctional (“DAWG ”) and functional as well as nonfunctional (“simgenome ”) DNA sequences from the twelve species of *Drosophila* described in [43] were produced with the DAWG [29] and simgenome [30] programs as described in [30] (both were parametrized based on Pecan alignments of *Drosophila* whole-genome alignments). Three of the simgenome alignments contained sequences of length zero and were removed from this analysis. FSA was run with the –exonerate option to use both anchors from the exonerate program as well as MUMs from MUMmer. FSA had the highest accuracy on the two simulated *Drosophila* datasets and only Pecan had higher accuracy on the mammalian dataset. Pecan consistently produced the most-sensitive aligments.

The simulated alignments of nonfunctional DNA sequences from nine mammals (human, chimp, baboon, mouse, rat, cat, dog, cow, and pig) were created by Blanchette et al. [28]. The simulated alignments of DNA from the twelve species of *Drosophila* described in [43] were created with two simulation programs, DAWG [29] and simgenome [30]. As described in [30], the simulated *Drosophila* genomic alignments were created by parameterizing the DAWG and simgenome programs using whole-genome alignments produced by Pecan for [43]. Although two authors (RKB and IH) of this manuscript contributed to the simgenome program, simgenome was developed prior to FSA and did not influence or contribute to the methodology described here for FSA.

FSA's strong performance on all three datasets of simulated long DNA sequences indicate that it is a useful and accurate tool for genomic alignment.

### Unrelated sequence

In order to further test the appropriateness of using popular alignment programs to detect homology between sequences, we conducted a large-scale random-sequence experiment. We generated datasets of random sequences to simulate unrelated protein, short DNA, and genomic (long) DNA sequences. The results, shown in Table 4 and Table 5, clearly demonstrate that while for most alignment programs, aligned sequences are not necessarily homologous, FSA leaves random sequences largely unaligned.

**Table 4:** Benchmarks against simulated unrelated protein and DNA sequences.

Program	Protein	DNA
AMAP	14%	n/a
ClustalW	97%	95%
DIALIGN	24%	17%
FSA	**4**%	**5**%
FSA (–maxsn)	21%	17%
MAFFT	83%	93%
MUMMALS	63%	n/a
MUSCLE	89%	80%
Probalign	44%	n/a
ProbCons	51%	77%
T-Coffee	63%	75%
SeqAn::T-Coffee	74%	78%

Large-scale random sequence tests indicate that for most alignment programs, aligned sequences are not necessarily homologous (table shows the fraction of random sequence aligned, calculated by taking a sum-of-pairs over pairwise alignments). Even when run in maximum-sensitivity mode (–maxsn), FSA aligned only a small fraction of the random sequence. We generated 50 datasets, each with 10 random sequences, and ran all programs with default parameters. Protein sequences were 300 aa in length and DNA sequences were 1,000 nt in length. Results reported for ProbCons on DNA sequences were obtained with ProbConsRNA.

**Table 5:** Benchmarks against simulated unrelated genomic DNA.

Program	Genomic DNA
CHAOS/DIALIGN	10%
ClustalW	96%
DIALIGN-TX	20%
FSA (–exonerate)	1%
FSA (–exonerate –maxsn)	4%
MAVID	17%
MLAGAN	30%
Pecan	1%
TBA	**0%**

Large-scale random sequence tests for genomic alignment programs. As in Table 4, table entries are the fraction of random sequence aligned, calculated by taking a sum-of-pairs over pairwise alignments. FSA aligns a small fraction of random genomic sequence in both its default and maximium-sensitivity (–maxsn) modes. TBA did not align a single base in these tests and was thus the best performer. As the three best-performing programs in this test, TBA, Pecan and FSA –exonerate, all use inexact sequence matches as anchors, the relative performance of these three programs can be explained by the stringency of the anchoring thresholds used: TBA uses the highest threshold by default, Pecan the next-highest and FSA the lowest. All three of these programs show good base-level specificity on the simulated alignments of Table 3, for which TBA has the highest specificity on one dataset and FSA on two. The random sequence tests consisted of 50 datasets, each with 10 random DNA sequences (uniform base distribution) of length 50 kb. All programs were run with default parameters. For genomic aligners that required a phylogenetic tree, we used the guide tree computed by ClustalW (rooted via the midpoint algorithm of the PHYLIP [64] retree program).

### Concordance between amino acid and nucleotide alignments

Biologists commonly align coding regions in both amino acid and nucleotide space, but there have been few studies of the effectiveness of alignment programs across the two regimes. We tested the consistency of alignment programs on coding sequence by aligning all 1,502 genes in *Saccharomyces cerevisiae* identified as having orthologs in the six related yeast species *S. paradoxus*, *S. mikatae*, *S. kudriavzevii*, *S. bayanus*, *S. castellii*, and *S. kluyveri* ([44]; this dataset was also analyzed in [5]). As shown in Table 6, alignments produced by FSA had higher concordance (0.943) than those produced by any other program. If a program produces alignments with low concordance between amino acid and nucleotide alignments, then at least one of the alignments must be incorrect (and quite possibly both are).

**Table 6:** Comparisons of alignments obtained in codon and amino acid space.

Program	Alignment similarity (average)
ClustalW	0.914
DIALIGN	0.912
FSA	**0.943**
FSA (–noanchored)	**0.952**
MAFFT	0.932
MUSCLE	0.915
ProbCons	0.902
T-Coffee	0.897
SeqAn::T-Coffee	0.905

We assessed the concordance between alignments obtained in nucleotide and amino acid space by aligning all 1,502 genes in *Saccharomyces cerevisiae* which have orthologs in the six related yeast species *S. paradoxus*, *S. mikatae*, *S. kudriavzevii*, *S. bayanus*, *S. castellii*, and *S. kluyveri* (this dataset was analyzed in [5]). Alignments produced by FSA, in both anchored and unanchored (–noanchored) modes, had the highest concordance. Alignment similarity between alignments computed in nucleotide and amino acid space was assessed by converting the amino acid alignment to the implied nucleotide alignment and computing the alignment similarity (the proportion of identical homology statements made by the alignments; see Text S1, “The mathematics of distance-based alignment” for details) between them. Alignments for ProbCons on nucleotide sequences were obtained with ProbConsRNA.

This simple test additionally indicates the effectiveness and robustness of FSA's query-specific learning. While very different learning procedures are used for amino acid and nucleotide sequence, the concordant alignments inferred by FSA indicate that our results are robust with respect to the details of the learning procedure.

### Ablation analysis of FSA's components

We conducted an ablation analysis of FSA's components to test the effectiveness of each component and ensure that they all contributed to the accuracy of the program. As indicated by the results in Tables 7–10, each optional component of FSA contributes to its accuracy.

**Table 7:** Ablation analysis of FSA on protein structural databases.

FSA options	BAliBASE 3	BAliBASE 3+fp	SABmark 1.65
	(Acc/Sn/PPV)	(Acc/Sn/PPV)	(Acc/Sn/PPV)
(default)	0.71/0.62/0.85	**0.75**/0.62/0.84	**0.59**/0.38/0.52
–fast	0.70/0.61/0.85	0.74/0.62/0.84	0.59/0.37/0.52
–nolearn	0.72/0.65/0.81	0.75/0.65/0.79	0.56/0.44/0.44
–refinement 0	0.70/0.61/0.85	0.74/0.61/0.84	0.59/0.37/0.52
–noindel2	0.70/0.61/0.85	0.74/0.60/0.84	0.59/0.38/0.52
–maxsn	**0.73**/0.68/0.76	0.74/0.68/0.72	0.52/0.45/0.39
–fast –maxsn	0.73/0.67/0.76	0.73/0.67/0.71	0.52/0.44/0.39
–nolearn –maxsn	0.73/0.68/0.74	0.70/0.68/0.67	0.49/0.47/0.37
–refinement 0 –maxsn	0.72/0.66/0.78	0.73/0.66/0.73	0.53/0.43/0.39
–noindel2 –maxsn	0.73/0.68/0.76	0.72/0.68/0.70	0.51/0.45/0.39

Ablation analysis of FSA on the protein benchmarks of Table 1 : Comparisons of the accuracies (Acc), sensitivities (Sn) and positive predictive values (PPV) of FSA with different components enabled or disabled. From top to bottom, FSA was run in default mode, –fast mode, with learning disabled, with iterative refinement disabled, and with 1 set (rather than 2 sets) of indel states; these options were then repeated for maximum-sensitivity mode (–maxsn). As made evident by the results (PPV) on the BAliBASE 3+fp and SABmark 1.65 datasets, query-specific learning helps FSA to distinguish homologous and non-homologous sequences. The above figures understate the utility of iterative refinement: while it generally has little effect on these small protein alignments, it occasionally dramatically reduces the number of small gaps and thereby improves the alignment accuracy.

**Table 8 pcbi-1000392-t008:** Ablation analysis of FSA on RNA structural databases.

FSA options	BRAliBase 2.1	FSA options	Consan mix80
	(Acc/Sn/PPV)		(Acc/Sn/PPV)
(default)	0.90/0.91/0.94		
–fast	0.90/0.91/0.94	–fast	0.77/0.74/0.92
–nolearn	0.91/0.92/0.93	–nolearn –fast	0.77/0.74/0.93
–refinement 0	0.90/0.91/0.93	–refinement 0 –fast	0.73/0.69/0.94
–noindel2	0.91/0.92/0.93	–noindel2 –fast	0.73/0.69/0.91
		–noanchored –fast	0.77/0.74/0.93
–maxsn	**0.91**/0.92/0.92		
–fast –maxsn	0.91/0.92/0.92	–fast –maxsn	0.78/0.78/0.86
–nolearn –maxsn	0.91/0.92/0.92	–nolearn –fast –maxsn	0.78/0.78/0.85
–refinement 0 –maxsn	0.90/0.91/0.93	–refinement 0 –fast –maxsn	0.74/0.70/0.92
–noindel2 –maxsn	0.91/0.92/0.92	–noindel2 –fast –maxsn	0.74/0.73/0.84
		–noanchored –fast –maxsn	**0.79**/0.79/0.85

Ablation analysis of FSA on the RNA benchmarks of Table 2 : Comparisons of the accuracies (Acc), sensitivities (Sn) and positive predictive values (PPV) of FSA with different components enabled or disabled. From top to bottom, FSA was run in default mode, –fast mode, with learning disabled, with iterative refinement disabled, with 1 set (rather than 2 sets) of indel states, and with anchored disabled; these options were then repeated for maximum-sensitivity mode (–maxsn). Iterative refinement is important for the large alignments of the mix80 dataset.

**Table 9 pcbi-1000392-t009:** Ablation analysis of FSA on simulated mammalian genomic DNA.

FSA options	Blanchette et al.
	(Acc/Sn/PPV)
(default)	0.53/0.32/0.93
–exonerate	0.83/0.77/0.94
–exonerate –minscore 50	**0.83**/0.78/0.94
–exonerate –refinement 0	0.82/0.76/0.93
–exonerate –noindel2	0.78/0.72/0.94

Ablation analysis of FSA on the simulated mammalian DNA of Table 3 : Comparisons of the accuracies (Acc), sensitivities (Sn) and positive predictive values (PPV) of FSA with different components enabled or disabled. We tested the effectiveness of components related to anchor annealing for aligning long sequences, including using anchors from MUMmer and exonerate and changing the minimum acceptable score for an exonerate anchor (the default is –minscore 100). These results clearly show that while using only MUMs for anchoring (the default mode) gives a high positive predictive value, inexact matches must be used to obtain high sensitivity on very long or distant nonfunctional sequences lacking the local constraints which give rise to MUMs across species in functional (e.g., coding) sequence.

**Table 10:** Ablation analysis of FSA on simulated unrelated protein and DNA sequences.

FSA options	Protein	DNA
(default)	4%	5%
–fast	4%	5%
–nolearn	13%	8%
–refinement 0	**3**%	**5**%
–noindel2	5%	10%
–maxsn	21%	17%
–fast –maxsn	22%	17%
–nolearn –maxsn	30%	16%
–refinement 0 –maxsn	19%	15%
–noindel2 –maxsn	27%	21%

Ablation analysis of FSA on the unrelated sequence benchmarks of Table 4 : Comparisons of the accuracies (Acc), sensitivities (Sn) and positive predictive values (PPV) of FSA with different components enabled or disabled. From top to bottom, FSA was run in default mode, –fast mode, with learning disabled, with iterative refinement disabled, and with 1 set (rather than 2 sets) of indel states; these options were then repeated for maximum-sensitivity mode (–maxsn). Query-specific learning helps to make FSA robust to non-homologous sequence.

The importance of each component depends strongly upon the alignment problem. The –fast heuristic for reducing the number of sequence pairs used to construct an alignment results in little loss of accuracy, at least on the benchmarks used in this paper (Tables 7 and 8). As indicated by the small and long RNA benchmarks (Table 8), iterative refinement is important for aligning many sequences and less so for small alignment problems. The anchor annealing procedure appears to be an effective heuristic for aligning long sequences. Anchoring with unique matches (MUMs) causes only a negligible loss of accuracy on the long RNA dataset (Table 8). However, results on simulated long DNA sequences (Table 9) demonstrate that inexact matches, such as those returned by exonerate, must be used during anchor annealing to obtain high sensitivity on very long or distant nonfunctional DNA sequences. Nonfunctional DNA lacks the local constraints which preserve exact matches across distant species in functional (e.g., coding) sequence. Query-specific learning is important for maintaining FSA's robustness to non-homologous sequence. While FSA aligned only 4% of random protein sequences in default mode, when run without learning it aligned 13% (Table 10), similar to the 14% aligned by AMAP (Table 4).

### Runtimes and parallelization

Biologists commonly perform alignments of hundreds or thousands of 16S ribosomal DNA sequences in order to elucidate evolutionary relationships and build phylogenetic trees. We performed alignments of prokaryotic 16S sequences to compare the speed of commonly-used programs (Table 11). MAFFT was the fastest method by an order of magnitude; MUSCLE and FSA were the next-fastest methods. Many alignment programs were unable to align these large datasets.

**Table 11:** Timing comparison of FSA and other methods on 16S sequences.

Program	100	200	300	400	500 seqs
ClustalW	1,194 s	4,147 s	9,110 s	16,187 s	27,755 s
DIALIGN	4,346 s	19,449 s	49,388 s	(fail)	(fail)
FSA –fast	1,513 s	3754 s	5,641 s	9,767 s	15,683 s
FSA –fast –noindel2 –refinement 0	638 s	1,495 s	2,467 s	3,604 s	5,154 s
MAFFT	**31 s**	**105 s**	**243 s**	**442 s**	**54 s**
MUSCLE	351 s	1,235 s	1,516 s	4,384 s	7,552 s
ProbConsRNA	16,319 s	(fail)	(fail)	(fail)	(fail)
T-Coffee	1,362 s	3,666 s	7,880 s	15,254 s	22,085 s
SeqAn::T-Coffee	3,024 s	(fail)	(fail)	(fail)	(fail)

Comparison of runtimes of FSA and other alignment methods when aligning 16S ribosomal sequences. MAFFT was faster than any other method by an order of magnitude; the next-fastest programs were MUSCLE and FSA. FSA can be made substantially faster by using a 3-state, rather than the default 5-state, HMM (with little loss of accuracy; see Table 8) and disabling iterative refinement. MAFFT was run with the –auto option, which presumably triggered a faster alignment mode on the 500 sequence dataset than was used for the datasets with fewer sequences. The designation “(fail)” means that a programs failed to align a dataset (generally due to out-of-memory errors). Timing results are from computers with 2.40 GHz CPUs and 2 GB of RAM. 16S sequences were obtained as a random slice of prokMSA from Greengenes [65] and had an average length of 1,450 nt.

The results in Table 12 and Table 13 demonstrate the effectiveness of FSA's parallelization mode. Parallelizing the pairwise comparisons dramatically reduces runtime: When running in –fast mode on a small cluster with 10 processors, FSA can align 500 16S sequences in 20% of the time required without parallelization.

**Table 12:** Timing comparison of FSA in regular and parallelized modes.

FSA options	100	200	300	500	1,000 seqs
FSA	6,407 s	27,534 s	—	—	—
FSA –parallelize 10	819 s	5,713 s	22,113 s	—	—
FSA –fast	1,650 s	3,781 s	6,207 s	12,249 s	—
FSA –fast –parallelize 10	201 s	513 s	924 s	2,511 s	15,179 s

Runtimes for FSA in regular, –fast and –parallelize modes when aligning the 16S sequences of Table 11 sequences in unanchored mode (–noanchored) with a 3-state HMM (–noindel2) and refinement disabled (–refinement 0). When running in –fast mode on a cluster with 10 processors (3.00 and 3.20 GHz; 8 GB of RAM), FSA can align 500 16S sequences in 20% of the time required without parallelization. The parallelized FSA was run on a cluster managed by the Condor batch queueing system [66]; nodes were connected by a 100 Mbps Ethernet network. Note that these runtimes are much slower than users can expect from default FSA usage, which uses anchoring for speed (Table 11); we used unanchored mode to make clear the benefits of parallelization.

**Table 13:** Timing comparison of FSA in parallelized mode with different numbers of processors.

	1	5	10	15	20 processors
100 seqs	1,650 s	365 s	214 s	135 s	105 s
200 seqs	3,781 s	889 s	506 s	385 s	355 s

Runtimes for FSA in –fast –parallelize P mode as a function of the number of processors P in the computer cluster with a 3-state HMM (–noindel2) and refinement disabled (–refinement 0). Sequences and cluster specifications are same as for Table 12.

## Discussion

In the Introduction we highlighted four design criteria which we emphasized in the development of FSA. The first goal was to find alignments with high expected accuracy, where an accurate alignment has minimal distance to the truth. This objective function is markedly different from both the maximum-likelihood approaches used by programs such as ClustalW and MUSCLE and the maximum expected sensitivity approaches used by programs such as ProbCons and Pecan. Note that while the objective function used in ProbCons is called “maximum expected accuracy” in the paper [37], it is actually a maximum expected sensitivity objective function, where there is no penalty for over-aligning sequence (c.f., the results shown in Table 4). Their objective function can be recovered as a special case of our approach by ignoring the gap probabilities in FSA's objective function (Text S1, “The mathematics of distance-based alignment”). FSA's explicit search for the most accurate, rather than most likely or most sensitive, alignment is what allows FSA to so dramatically outperform most other programs on tests on unrelated sequence (Table 4).

We believe that this accuracy criterion, which gives equal weight to the correctness of all sequence positions, is a natural measure of alignment quality. Downstream analyses, such as phylogenetic reconstruction and evolutionary constraint analysis, are increasingly using indels in addition to homologous characters for more accurate estimation (e.g., [45],[46]). Thus, it is important that alignments be as “evolutionarily correct” as possible [47], which is the purpose of the accuracy criterion.

FSA's strong performance under the accuracy criterion is due to techniques such as its iterative refinement as well as its explicit attempt to maximize the expected accuracy; programs which explicitly seek to maximize an objective function of the posterior probabilities of character alignment, such as ProbCons or Probalign, could instead seek to maximize the expected accuracy described here and, as a likely result, increase their robustness to non-homologous sequence. However, while we believe that the expected accuracy is a biologically-sensible objective function, it may not be appropriate if the user desires the most sensitive alignment. While FSA can produce the most-sensitive RNA alignments, other programs can produce more sensitive alignments of proteins and genomic sequence, albeit generally at the cost of a tendency to align non-homologous sequence (Table 4).

The second goal was to create alignments which are robust to evolutionary distances and different functional constraints on patterns of molecular evolution. FSA's unsupervised query-specific learning for parameter selection frees the user from unknown systematic biases implicitly introduced by using an alignment program whose parameters were trained on a dataset whose statistics may not reflect those of the sequences to be aligned. For example, it is traditionally challenging to align sequences with unusual base composition, but FSA can easily handle such alignments by automatically learning appropriate parameters. As indicated by our ablation analysis, query-specific learning further increases FSA's robustness to non-homologous sequences beyond that offered by the minimum-distance objective function alone.

We believe that FSA's unsupervised query-specific learning is the first time a multiple alignment program has been capable of dynamically learning a complete parameterization, wherein parameters can vary for each pair of sequences to be compared, on the fly. This learning method is related to the “pre-training” option in ProbCons, which permits users to learn different models for families of homologous sequences, but does not permit parameterizations to vary between sequence pairs. We also note that the MORPH program for pairwise alignment of sequences with *cis*-regulatory modules learns model transition parameters from data [48]. While supervised training on curated data can give superior performance on test sets which are statistically-similar to the training data, the practical alignment problems encountered everyday by biologists do not fit into this rigid problem setup. Query-specific learning consistently gives reasonable performance.

The third and fourth goals, to develop a single, modular program which can address all typical alignment problems encountered by biologists, are naturally achieved within FSA's architecture. While almost all alignment programs are designed to either align many short sequences or a few long sequences, we have demonstrated that it is feasible to create a single program which can address both situations. This is made practical by FSA's modular nature, where the statistical model for pairwise comparisons, the anchoring scheme for finding homology between long sequences, and the sequence annealing procedure are entirely separate and can be individually modified and improved. For example, the parallelization of FSA was designed and developed with minimal changes to the rest of FSA's code base. Similarly, while FSA's basic anchoring relies only on exact matches from MUMmer, the anchoring scheme was easily extended to incorporate inexact matches from exonerate [17] and alignment constraints from Mercator [22]. In fact, this flexibility permits FSA to incorporate almost any sources of potential homology information, from predicted transcription factor binding sites to entire gene models. One natural extension of FSA's approach is to models of RNA alignment which take structure into account. The program Stemloc-AMA [49] uses a model of the pairwise evolution of RNA secondary structure in conjunction with the sequence annealing algorithm to create accurate multiple alignments of structured RNAs. By using Stemloc-AMA's probabilistic model rather than a Pair HMM and taking advantage of techniques such as query-specific learning, FSA could sum over possible pairwise structural alignments in order to get better estimates of posterior probabilities of character alignment.

FSA is a statistical alignment program insofar as it uses an explicit statistical model of alignments and a probabilistic objective function for optimization, but as discussed in “Theoretical justification of distance-based alignment” (Text S1), it also is a distance-based approximation to the “phylogenetic alignment” models of alignments on trees [8]–[11], [50]–[52]. While traditional phylogenetic alignment algorithms are currently too computationally-expensive to use on datasets of more than a few sequences, FSA's distance-based method allows biologists to use the sophisticated tools of statistical alignment algorithms on practical problems. Furthermore, while we have not addressed the phylogenetic aspects of FSA in detail in this paper, our methods can be adapted to incorporate a complete phylogenetic model (Text S1, “The mathematics of distance-based alignment”). However, we believe that FSA's current approach, which is agnostic to phylogeny, offers many practical advantages for common genomics analyses. For example, because FSA uses a sum-of-pairs objective function, there is no guide tree to potentially bias downstream phylogenetic reconstructions based on the alignment. Similarly, while most genomic alignment programs require a species tree to perform the alignment, our phylogeny-free approach will be avoid potential biases introduced by using a single species tree to align regions which may have undergone recombination.

## Supporting Information

Text S1 Supplementary Information
(0.23 MB PDF)
