# Supplementary material for: Fast Statistical Alignment
Source: PLoS Comput Biol. 2009 May 29;5(5):e1000392. doi: 10.1371/journal.pcbi.1000392 (PMC2684580; doi:10.1371/journal.pcbi.1000392)
Supplement: Text S1 — Supplementary Information (0.23 MB PDF) [file pcbi.1000392.s001.pdf]

# Fast Statistical Alignment: Text S1

Robert K. Bradley[1,*], Adam Roberts[2], Michael Smoot[3], Sudeep Juvekar[2], Jaeyoung Do[4], Colin Dewey[4,5], Ian Holmes[6], Lior Pachter[1]

**1** Department of Mathematics and Department of Molecular & Cellular Biology, University of California, Berkeley, CA 94720, USA

**2** Department of EECS, University of California, Berkeley, CA 94720, USA

**3** Department of Bioengineering, University of California, San Diego, CA 92093, USA

**4** Department of Computer Sciences, University of Wisconsin, Madison, WI 53706, USA

**5** Department of Biostatistics & Medical Informatics, University of Wisconsin, Madison, WI 53706, USA

**6** Department of Bioengineering, University of California, Berkeley, CA 94720, USA

∗ E-mail: rbradley@berkeley.edu

# Contents

**1 Requirements and Installation** **3**

**2 Webserver** **4**

**3 GUI** **5**

**4 Parallelization** **8**

**5 Complete list of options** **9**

**6 Utility programs** **14**

**7 The mathematics of distance-based alignment** **16**

# 1 Requirements and Installation

FSA is released under the GNU General Public License (http://www.gnu.org/copyleft/gpl.html). The source code can be downloaded from its SourceForge project homepage, http://fsa.sourceforge.net/, and should build and run smoothly on most *NIX machines, including Mac OS X and popular distributions of Linux. The program is compiled by navigating to the FSA root directory and typing the standard configure, make, make install. Use the configure script to specify installation options, etc. In order to align long DNA sequences, FSA requires that MUMmer [1] be installed. It is recommend that exonerate [2] is installed as well.

If you wish to run FSA in parallelized mode, you must have a PostgreSQL [3] database available and a computer cluster managed by Condor [4].

## 2 Webserver

Alignment jobs can be submitted to the `FSA` webserver at http://orangutan.math.berkeley.edu/fsa/. Due to computational constraints, large alignment jobs involving hundreds of sequences or very long sequences will not be completed.

## 3 GUI

In order to use `FSA`'s GUI to interact with alignments, run `FSA` with the --gui option. If aligning a sequence file myseqs.fasta as

```
fsa --gui myseqs.fasta
```

then the GUI is subsequently invoked as

```
java -jar display/mad.jar myseqs.fasta
```

If you wish to also color an alignment myseqs.mfa of these sequences, invoke the GUI as

```
java -jar display/mad.jar myseqs.fasta myseqs.mfa
```

`FSA`'s GUI can color alignments according to estimates of five different measures of alignment quality. A character $x_i$ in a multiple alignment $\mathcal{A}^*$ can be colored according to one of the following measures, all of which are normalized to $[0, 1]$:

**Accuracy.** The expected accuracy with which $x_i$ is aligned to other characters or gaps in the column.

$$\text{Accuracy}\,(x_i) = 2 \cdot \sum_{Y:\, x_i \sim y_j \in \mathcal{A}^*} \mathbb{P}(x_i \sim y_j | X, Y) + \sum_{Y:\, x_i \sim - \in \mathcal{A}^*} \mathbb{P}(x_i \sim - | X, Y) \Big/ \left(2 \cdot |\{Y : x_i \sim y_j \in \mathcal{A}^*\}| + |\{Y : x_i \sim - \in \mathcal{A}^*\}|\right)$$

This definition is analogous to the definition of expected alignment accuracy $\mathbb{E}\left[Acc\left(\mathcal{A}^*\right)\right]_{\mathbb{P}(\mathcal{A}|data)}$ given previously, but restricted to a single character $x_i$ in the alignment. The denominator ensures that $\text{Accuracy}\,(x_i)$ is normalized to $[0, 1]$.

**Sensitivity.** The expected sensitivity with which $x_i$ is aligned to other characters in the column.

$$\begin{aligned}\text{Sensitivity}\,(x_i) &= \sum_Y \mathbb{E}\left[\text{tp}\right]_{\mathbb{P}(\mathcal{A}|X,Y)} \Big/ \sum_Y \mathbb{E}\left[\text{tp} + \text{fn}\right]_{\mathbb{P}(\mathcal{A}|X,Y)} \\ &= \sum_{Y:\, x_i \sim y_j \in \mathcal{A}^*} \mathbb{P}(x_i \sim y_j | X, Y) \Big/ \sum_Y \sum_{y_j \in Y} \mathbb{P}(x_i \sim y_j | X, Y)\,,\end{aligned}$$

where tp = true positives and fn = false negatives. Note that this is an approximation of $\mathbb{E}\left[\mathrm{tp}/\left(\mathrm{tp}+\mathrm{fn}\right)\right]_{\mathbb{P}(\mathcal{A}|X,Y)}$.

**Specificity.** The expected specificity (positive predictive value) with which $x_i$ is aligned to other characters in the column.

$$\begin{aligned}\text{Specificity}\left(x_i\right) &= \sum_Y \mathbb{E}\left[\mathrm{tp}\right]_{\mathbb{P}(\mathcal{A}|X,Y)} \Bigg/ \sum_Y \mathbb{E}\left[\mathrm{tp}+\mathrm{fp}\right]_{\mathbb{P}(\mathcal{A}|X,Y)} \\ &= \sum_{Y:\, x_i\sim y_j\in\mathcal{A}^*} \mathbb{P}(x_i\sim y_j|X,Y) \Bigg/ \left|\{Y:\, x_i\sim y_j\in\mathcal{A}^*\}\right| ,\end{aligned}$$

where tp = true positives and fp = false positives. Note that this is an approximation of $\mathbb{E}\left[\mathrm{tp}/\left(\mathrm{tp}+\mathrm{fp}\right)\right]_{\mathbb{P}(\mathcal{A}|X,Y)}$.

**Certainty.** The certainty with which $x_i$ is aligned correctly (whether there is a good alternate choice) to other characters or gaps in the column:

$$\begin{aligned}\text{Certainty}\left(x_i\right) = 2\cdot &\sum_{Y:\, x_i\sim y_j\in\mathcal{A}^*} \mathbb{P}(x_i\sim y_j|X,Y) + \sum_{Y:\, x_i\sim -\in\mathcal{A}^*} \mathbb{P}(x_i\sim -|X,Y) \\ &\Bigg/ \left(2\cdot \sum_{Y:\, x_i\sim y_j\in\mathcal{A}^*} \operatorname*{altmax}_{y_j} \mathbb{P}(x_i\sim y_j|X,Y)\right. \\ &\qquad \left. + \sum_{Y:\, x_i\sim -\in\mathcal{A}^*} \operatorname*{altmax}_{y_j} \mathbb{P}(x_i\sim y_j|X,Y)\right) ,\end{aligned}$$

where $\mathrm{altmax}_{y_j}\, \mathbb{P}(x_i\sim y_j|X,Y)$ is the probability of the second-best choice for aligning $x_i$ to either a character $y_j$ or a gap in $Y$. As defined above, this measure can range from $[0,\infty)$; we normalize it to $[0,1]$ for display in the GUI by taking

$$\text{Certainty}\left(x_i\right) \leftarrow \begin{cases} 0 & \text{if Certainty}\left(x_i\right) < 1 \\ \log \text{Certainty}\left(x_i\right)/\log 5 & \text{if } 0 \leq \text{Certainty}\left(x_i\right) \leq 5 \\ 1 & \text{if Certainty}\left(x_i\right) > 5 \end{cases}$$

**Consistency.** The consistency of the many pairwise comparisons used to construct the multiple alignment and the implied optimality of the alignment of $x_i$ to other characters or gaps in the column.

$$\begin{aligned}\text{Consistency}\,(x_i) = 2 \cdot \sum_{Y:\, x_i \sim y_j \in \mathcal{A}^*} \mathbb{P}(x_i \sim y_j | X, Y) + \sum_{Y:\, x_i \sim - \in \mathcal{A}^*} \mathbb{P}(x_i \sim - | X, Y) \\ \Bigg/ \quad \Bigg( 2 \cdot \sum_{Y:\, x_i \sim y_j \in \mathcal{A}^*} \max_{y_j} \mathbb{P}(x_i \sim y_j | X, Y) \\ + \sum_{Y:\, x_i \sim - \in \mathcal{A}^*} \max_{y_j} \mathbb{P}(x_i \sim y_j | X, Y) \Bigg) \,,\end{aligned}$$

where $\max_{y_j} \mathbb{P}(x_i \sim y_j | X, Y)$ is the probability of the best choice for aligning $x_i$ to either a character $y_j$ or a gap in $Y$. The consistency measure is related to the certainty measure, but note that it is already normalized to $[0, 1]$.

# 4 Parallelization

Use the --parallelize option to parallelize the pairwise comparison step of `FSA`. For example, to align the sequences in the file myseqs.fasta with $N$ processors, invoke `FSA` as:

```
fsa --parallelize N myseqs.fasta
```

When run with these arguments, `FSA` will automatically create a `Condor`-specific submit file and submit $N$ jobs to your cluster.

FSA can also use a database to store pairwise posterior probabilities and initial weights of the null alignment. To use a database, you must provide the --hostaddr and --dbname options. Other database connection options include --port, --username and --password. For the example above, but with a database, invoke `FSA` as:

```
fsa --parallelize N --hostaddr [DB hostname/IP address] --dbname [DB name] myseqs.fasta
```

The --noannealing option may be used to disable the sequence annealing step. This option can be used when you only wish to compute pairwise probabilities and store them in a database. To align sequences with data already in a database, run `FSA` with --noposteriors and a combination of db connection options:

```
fsa --noposteriors --hostaddr [DB host name/IP address] --dbname [DB name] myseqs.fasta
```

When using `FSA` with a database, you can also restrict the maximum amount of RAM used during sequence annealing with the --db-maxram option (the --maxram option only affects the amount of RAM used when making pairwise comparisons between sequences). Without this option, `FSA` will load all necessary data into memory before beginning sequence annealing for performance reasons.

# 5 Complete list of options

FSA attempts to guess appropriate settings for the input sequences based on the sequence composition (nucleotide or protein), average sequence length, and number of input sequences. You can get fine-grained control over its inference procedures with the command-line options described below. Use the --help flag to see descriptions of all options.

Any Boolean option (e.g., --learngap) can be disabled by prepending --no (e.g., --nolearngap).

**Logging options.** The progress of the algorithm can be assessed by adjusting the log level as --log [0-10]. Log level --log 7 reports progress of the dynamic programming and sequence annealing procedures and is recommended for big alignment jobs. Log level --log 6 reports progress of anchoring and is recommended when aligning very long sequences.

**Output options.** FSA produces multi-FASTA (MFA) format alignments by default. Use --stockholm to produce Stockholm-format alignments instead. FSA's Stockholm-format alignments are annotated with per-column accuracy information as well as a single accuracy estimate for the entire alignment.

You can record and subsequently view all intermediate alignments produced by the sequence annealing process. Use --gui to record them formatted for the Java interactive GUI. This option is also used to record the statistical model estimated by FSA in order to graphically view the expected accuracy of the alignment.

Use --write-params and --write-posteriors to record the learned emission parameters (substitution matrices) and pairwise posterior probability matrices to disk. See Section 6 for information on how to view these graphically.

**Parallelization options.** See Section 4.

**Database options.** See Section 4.

**Pair HMM model options.** If desired, FSA can align coding nucleotide sequence by first translating the sequences, aligning the resulting protein sequences, and then displaying the corresponding nucleotide-level alignment. Use --nucprot to invoke this behavior.

Use --noindel2 to use a Pair HMM with 1 set, rather than 2 sets, of indel states. The transition probabilities of the model can be set directly with the options illustrated in Figure 1. The substitution model for nucleotide or amino acid sequence can be chosen with the --model option. For DNA or RNA, the model can be either the Jukes-Cantor [5] or Tamura-Nei [6] model (default is Tamura-Nei). These models are further parameterized with the --time, --alphar, --alphay and --beta options. Protein sequences use the BLOSUM62 substitution matrix transformed such that the equilibrium distribution is equal to the empirical distribution over amino acids in the input sequences.

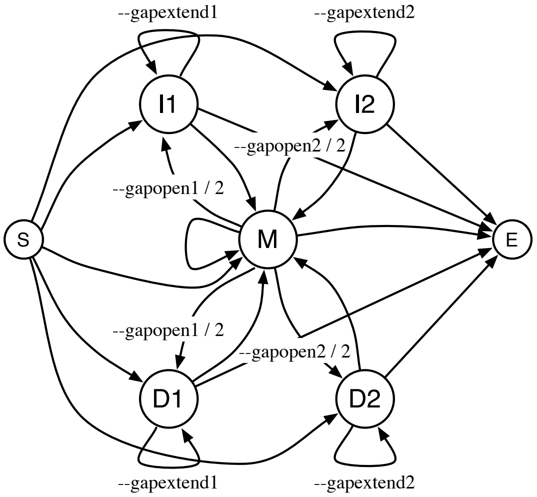


**Figure 1. The default 5-state Pair HMM used by** FSA with transitions labeled by the corresponding command-line options. FSA can optionally use a three-state HMM, which has only one set of Insert (I) and Delete (D) states; use --noindel2 to trigger this option.

Unless specified otherwise, FSA will use the corresponding transition and emission probabilities (whether chosen automatically or specified via command-line options) as seeds for the query-specific learning algorithm.

**Parameter estimation options.** FSA's query-specific learning uses unsupervised EM to learn appro-

priate parameters for each pair of input sequences. The --learngap, --learnemit-bypair and --learnemit-all options control whether FSA estimates transition probabilities and emission probabilities. If --learnemit-bypair is set, then FSA learns a separate emission distribution for each pair of sequences (default for nucleotide sequence); if --learnemit-all is set then FSA learns a single emission distribution for all sequences (default for amino acid sequence).

The --nolearn options disables all learning. In this case FSA uses the default ProbCons parameters.

The Dirichlet transition and emission regularization scales can be adjusted with --regularization-gapscale and --regularization-emitscale and regularization can be turned off entirely with --noregularize. The emission regularization scales correspond to the total number of pseudocount emissions because the seed distribution for pseudocount calculation (taken to be the seed emission parameters) is normalized to 1. The emission regularization scales are equal to the number of free parameters in a symmetric pair emission matrix, $4 \cdot (4-1)/2 + 4 = 10$ for nucleotides and $20 \cdot (20-1)/2 + 20 = 210$ for amino acids. We have observed that FSA's learning is insensitive to the transition regularization scale (qualitatively because there are many transition counts for typical pairs of sequences, thereby dwarfing the effects of the weak regularizer), which is set to 3 by default.

By default EM terminates when the log-likelihood increases by less than 10% (--mininc 0.1) and a maximum of three rounds of EM are permitted (--maxrounds 3).

The minimum amount of sequence data for learning transition and emission probabilities can be controlled with --mingapdata and --minemitdata. By default the minimum data for learning emission probabilities corresponds approximately to two DNA or RNA sequences of length 60 nucleotides (--minemitdata 60) or four protein sequences of length 266 amino acids (--minemitdata 1596), corresponding to observing each of the independent parameters of a substitution matrix four times.

**Multiple alignment options: sequence annealing.** The number of rounds of iterative refinement is controlled with --refinement. FSA can be run in maximum-sensitivity mode with --maxsn. For users wanting finer-grained control over the sensitivity/specificity trade-off, the gap factor can be specified with --gapfactor (the gap factor is explained in the main text). The default is --gapfactor 1, which corresponds

to the case where sequence annealing stops aligning characters when the probability that a character is aligned is equal to the probability that it is unaligned (aligned to a gap). The maximum-sensitivity mode is equivalent to using --gapfactor 0; values $> 1$ give higher specificity. Dynamic weighting can be disabled with --nodynamicweights, although this is generally not recommended (see the main text for a description of weighting).

**Alignment speedup options: many sequences.** The user can control the fraction or total number of pairwise comparisons made when building a multiple alignment with the --alignment-fraction and --alignment-number options. The number of comparisons used when aligning $N$ sequences can lie between $(N - 1)$ and $N \cdot (N - 1)/2$; as discussed in the main text, we observe good performance even when only a subset of pairs are used.

The number of pairwise comparisons used for parameter learning when --learnemit-all (the default for proteins) is enabled can be similarly controlled with --learning-fraction and --learning-number. Generally fewer pairwise comparisons are necessary for effective parameter learning than for constructing a multiple alignment.

The --fast option uses presets which are appropriate for aligning many sequences. It sets --alignment-fraction to the Erdös-Rényi threshold probability scaled by a factor of 5 and sets --learning-fraction to the threshold scaled by a factor of 2. As suggested by the results in the main text, the heuristics invoked by --fast are effective for difficult alignment problems.

The --refalign option aligns all sequences to a single reference sequence (taken to be the first sequence in the input file). No other pairwise comparisons are performed (so a total of $(N - 1)$ comparisons are made).

**Alignment speedup options: long sequences (`MUMmer`).** Anchoring can be turned on with the --anchored option and similarly disabled with --noanchored. By default, anchoring is turned on for nucleotide sequences longer than 200 nucleotides and disabled otherwise. Translated anchoring in protein space is invoked with --translated. The minimum length of anchors, controlled with --minlen, is 10 for nucleotide sequence and 7 for amino acid sequence (for anchoring protein sequences or translated anchoring of nu-

cleotide sequence). Because `MUMmer` reports only exact matches, by default `FSA` concatenates adjacent parallel anchors which are separated by at most 2 mismatches; this can be controlled with --maxjoinlen.

**Alignment speedup options: long sequences (`exonerate`).** `FSA` can use the `exonerate` program [2] to effectively obtain anchors between distant species, for which there are few exact matches. Use --exonerate to trigger anchor-finding with `exonerate` as well as `MUMmer`. By default `FSA` only uses candidate alignments with scores $\geq 100$; change this with --minscore. Use --softmasked to tell `exonerate` that the input sequences are softmasked.

**Alignment speedup options: long sequences (`Mercator`).** `FSA` can use the constraint maps produced by the homology-mapping program `Mercator` [7] to constrain the multiple alignment. Use --mercator to specify the `Mercator` constraint file.

**Memory savings.** `FSA`'s maximum memory consumption during inference can be controlled with --maxram. If the total RAM available can be read from system information (which `FSA` can do for BSD/Darwin and Linux systems), then --maxram is set by default to 85% of the total RAM. If an inference step requires more than the maximum memory allowed, then `FSA` leaves those sequences (or parts of sequences) unaligned.

The speed and memory consumption of each pairwise comparison can be reduced by "banding" the dynamic programming (DP) matrix with --bandwidth, thereby constraining the algorithm to only consider aligning characters $x_i$ and $y_j$ if $|i - j| <$ the bandwidth. Banding can be very effective but should be used with caution.

`FSA` uses a sparse representation of posterior probabilities, in which only posterior probabilities of character alignment greater than a cutoff are stored. By default this cutoff is 0.01; it can be increased with --minprob to reduce memory usage, although this is generally not recommended.

# 6 Utility programs

FSA is distributed with utility programs for viewing log files, manipulating and comparing alignments, estimating the accuracies of alignments under its statistical model, and viewing the inferred alignment posterior probabilities.

**Viewing log files.** FSA uses code from Ian Holmes's dart library [8] for logging. The script dartlog.pl can be used to color and follow log files as they are written by FSA.

**Comparing alignments.** The script cmpalign.pl compares two alignments and computes the corresponding accuracy, sensitivity and positive predictive value. This script uses the general-purpose Perl packages Stockholm and Stockholm::Database for parsing alignments, derived from the packages of the same names in the dart library [8]. They have been modified to parse more alignment formats (Stockholm, multi-FASTA, ClustalW and MSF).

**Working with alignments.** The program prot2codon finds the codon-level (nucleotide) alignment implied by a given alignment at the amino acid level.

The program gapcleaner implements the minimal chain decomposition described in the main text to find the most parsimonious ordering of indel events in an alignment.

**Working with whole-genome alignments.** FSA comes with programs for working with whole-genome alignments produced by FSA in conjunction with the homology-mapping program Mercator [7]:

- slice_mercator_alignment: Extract a subalignment from a FSA–Mercator whole-genome alignment.
- isect_mercator_alignment_gff: Extract subalignments from a FSA–Mercator whole-genome alignment for the features in a GFF file.
- map_coords: Map coordinates for one genome to another genome using the base-level homology mapping implied by a FSA–Mercator whole-genome alignment.

- `map_gff_coords`: Map coordinates for the features in a GFF file for one genome to another genome using the base-level homology mapping implied by a `FSA–Mercator` whole-genome alignment.

**Using `FSA`'s statistical model.**

- `accuracy.pl`: Compute the expected accuracy, sensitivity, positive predictive value, certainty and consistency for an alignment under `FSA`'s statistical model. Use in conjunction with the --gui option for `FSA`.

- `seqdotplot.pl`: Create colored dotplots of the pairwise alignment posterior probabilities. Use in conjunction with the --write-params option for `FSA`.

`seqdotplot.pl` is from the `dart` library.

# 7 The mathematics of distance-based alignment

**The POSET view of alignments.** `FSA` defines an alignment to be a set of homology statements between characters. This definition of an alignment is expressed mathematically as a partially ordered set (POSET) [9], which is equivalent to viewing an alignment as a Directed Acyclic Graph (DAG) as described in [10] and implemented in the `POA` alignment program.

A biologically-intuitive metric on alignments (introduced in [11]) emerges naturally from this definition. We define the distance $d(\mathcal{A}^*, \mathcal{A})$ between two alignments to be just the number of characters for which they make different homology statements. For two sequences $X$ and $Y$ of lengths $L_X$ and $L_Y$, the distance between two alignments is just $d(\mathcal{A}^*, \mathcal{A}) = L_X + L_Y - Sim(\mathcal{A}^*, \mathcal{A})$, where the similarity measure $Sim(\mathcal{A}^*, \mathcal{A})$ is the number of characters for which $\mathcal{A}^*$ and $\mathcal{A}$ make identical homology statements. The distance is therefore computed as:

$$d(\mathcal{A}^*, \mathcal{A}) = L_X + L_Y - \Bigg[2 \cdot \sum_{i,j:\, x_i \sim y_j \in \mathcal{A}^*} \mathbf{1}\{x_i \sim y_j \in \mathcal{A}\} + \sum_{i:\, x_i \sim - \in \mathcal{A}^*} \mathbf{1}\{x_i \sim - \in \mathcal{A}\} + \sum_{j:\, - \sim y_j \in \mathcal{A}^*} \mathbf{1}\{- \sim y_j \in \mathcal{A}\}\Bigg] . \quad (1)$$

This distance $d(\mathcal{A}^*, \mathcal{A})$ between alignments also has a natural mathematical expression. An alignment $\mathcal{A}$ induces a hierarchy on (or partition of) the set $\mathbb{X}$ of all characters in the aligned sequences. The distance between two alignments is just the size of the symmetric difference of the two corresponding hierarchies induced on the set $\mathbb{X}$, $d(\mathcal{A}^*, \mathcal{A}) = \mu(\mathcal{A}^* \,\Delta\, \mathcal{A})$, where the size $\mu$ of the symmetric difference is the cardinality of the set $\mathbb{Y}$ which the resulting symmetric difference is defined over.

**Inferring indel events.** As discussed earlier, the POSET definition of alignments does not attach significance to gap orderings which do not affect the homology specifications of the alignment. `FSA` outputs the global alignment with the minimum number of "gap openings" across the individual sequences.

Finding this minimum-indel global alignment corresponds to finding a minimal-chain decomposition of the alignment POSET. Such a minimal-chain decomposition (Dilworth decomposition) of an arbitrary

POSET can be found in time cubic in the number of nodes [12]. Thanks to the special structure of sequence graphs, however, by Theorem 7.1 `FSA` can find this minimum-indel global alignment in time linear in the number of vertices and edges in the graph with a depth-first search.

**Theorem 7.1** *The minimal-chain decomposition of a POSET which arises from a linear extension of the POSET can be found in time linear in the number of nodes by a depth-first search of the corresponding DAG.*

**Proof** Let $M(\mathcal{P})$ be the minimal number of chains in a chain decomposition arising from a linear extension of a POSET $\mathcal{P}$ with elements $\{x\}$. Note that $M(\mathcal{P}) \geq I(\mathcal{P})$, where

$$I(\mathcal{P}) = \sum_{x:\, indegree\,(x)>1} indegree\,(x)\,.$$

Here we are treating the POSET as a DAG. More precisely, the DAG represents the transitive reduction of the partial order, wherein nodes of the DAG represent one or more characters of the alignment and edges of the DAG represent the ordering imposed by the sequences.

Now consider imposing a topological ordering on the corresponding DAG according to the reverse finishing times of a postorder traversal of the graph. This topological sort, consisting of a sequence of chains of the POSET, terminates each chain when the depth-first search reaches a vertex $x$ of the graph for which $indegree(x) > 1$. The topological sort obtained by a postorder traversal therefore has exactly $I(\mathcal{P})$ chains. As $M(\mathcal{P}) \geq I(\mathcal{P})$, this is a minimal chain decomposition. ▮

**The objective function.** `FSA` seeks to find the alignment with the minimum expected distance to the truth, where the distance $d\,(\mathcal{A}^*, \mathcal{A})$ between two alignments is defined in Equation 1 as the number of characters for which they make different homology statements. The (pairwise) alignment with the

minimum expected distance to the truth is therefore

$$\begin{aligned}\mathcal{A}_{optimal} &= \underset{\mathcal{A}^*}{\operatorname{argmin}}\ \mathbb{E}\left[d\left(\mathcal{A}^*, \mathcal{A}\right)\right]_{\mathbb{P}(\mathcal{A}|X,Y)} \\ &= \underset{\mathcal{A}^*}{\operatorname{argmin}}\ \mathbb{E}\left[L_X + L_Y - Sim\left(\mathcal{A}^*, \mathcal{A}\right)\right]_{\mathbb{P}(\mathcal{A}|X,Y)} \\ &= \underset{\mathcal{A}^*}{\operatorname{argmax}}\ \mathbb{E}\left[Sim\left(\mathcal{A}^*, \mathcal{A}\right)\right]_{\mathbb{P}(\mathcal{A}|X,Y)}\ .\end{aligned} \tag{2}$$

The alignment with the minimum expected distance to the truth is therefore equivalent to the alignment with the maximum expected similarity to the truth. This gives a natural measure of alignment accuracy, where we define alignment accuracy as the similarity to the truth and the expected alignment accuracy as the expected similarity to the truth,

$$\begin{aligned}Acc\left(\mathcal{A}^*\right) &= Sim\left(\mathcal{A}^*, \text{truth}\right)/\left(L_X + L_Y\right) \\ \mathbb{E}\left[Acc\left(\mathcal{A}^*\right)\right]_{\mathbb{P}(\mathcal{A}|X,Y)} &= \mathbb{E}\left[Sim\left(\mathcal{A}^*, \mathcal{A}\right)\right]_{\mathbb{P}(\mathcal{A}|X,Y)}/\left(L_X + L_Y\right).\end{aligned} \tag{3}$$

We divide by $(L_X + L_Y)$ to normalize the accuracy to the interval $[0, 1]$. We will speak equivalently of finding the alignment with the highest expected similarity to the truth and the highest expected accuracy.

The posterior probabilities over alignments $\mathbb{P}(\mathcal{A}|X, Y)$ used in the optimization are given by `FSA`'s statistical model (a Pair HMM). Recalling that $Sim\left(\mathcal{A}^*, \mathcal{A}\right)$ is the number of characters for which $\mathcal{A}^*$ and $\mathcal{A}$ make identical homology statements, we can express the optimal alignment by taking the expectation of Equation 1 and using the posterior probabilities that characters are aligned or gapped (these are

computed with the Forward-Backward algorithm):

$$
\begin{aligned}
\mathcal{A}_{optimal} &= \operatorname*{argmax}_{\mathcal{A}^*} \mathbb{E}\left[Sim\left(\mathcal{A}^*, \mathcal{A}\right)\right]_{\mathbb{P}(\mathcal{A}|X,Y)} \\
&= \operatorname*{argmax}_{\mathcal{A}^*} \sum_{\mathcal{A}} \left[\mathbb{P}\left(\mathcal{A}|X,Y\right) Sim\left(\mathcal{A}^*, \mathcal{A}\right)\right] \\
&= \operatorname*{argmax}_{\mathcal{A}^*} \left[2 \cdot \sum_{i,j:\, x_i \sim y_j \in \mathcal{A}^*} \mathbb{P}(x_i \sim y_j|X,Y) \right. \\
&\qquad \left. + \sum_{i:\, x_i \sim - \in \mathcal{A}^*} \mathbb{P}(x_i \sim -|X,Y) + \sum_{j:\, - \sim y_j \in \mathcal{A}^*} \mathbb{P}(- \sim y_j|X,Y) \right] ,
\end{aligned} \tag{4}
$$

FSA extends this definition of an optimal pairwise alignment to an optimal multiple alignment by taking sum-of-pairs over all sequences (see “Theoretical justification of distance-based alignment” for a derivation of the approximations involved therein).

FSA allows the user to control the sensitivity/specificity trade-off of the method by introducing a gap factor gf into the objective function (Equation 4) being optimized,

$$
\begin{aligned}
\mathcal{A}_{optimal} = \operatorname*{argmax}_{\mathcal{A}^*} & \left[2 \cdot \sum_{i,j:\, x_i \sim y_j \in \mathcal{A}^*} \mathbb{P}(x_i \sim y_j|X,Y) \right. \\
& \left. +\mathrm{gf} \cdot \left( \sum_{i:\, x_i \sim - \in \mathcal{A}^*} \mathbb{P}(x_i \sim -|X,Y) + \sum_{j:\, - \sim y_j \in \mathcal{A}^*} \mathbb{P}(- \sim y_j|X,Y) \right) \right] ,
\end{aligned}
$$

A gap factor $\mathrm{gf} = 1$ corresponds to optimizing the expected accuracy, in which case we stop aligning characters when the probability that a character is aligned is equal to the probability that it is unaligned (aligned to a gap); a lower gap factor emphasizes sensitivity and a higher gap factor emphasizes specificity. By default FSA uses $\mathrm{gf} = 1$.

**A steepest-ascent algorithm.** The sequence annealing algorithm greedily attempts to maximize the objective function given in Equation 4, where the posterior probabilities of alignment are given by FSA's statistical model. Because exact optimization is tractable only for two (short) sequences, sequence annealing depends on effective heuristics for optimizing this function.

Sequence annealing begins with the null alignment (all characters unaligned) and iteratively aligns single columns until a stopping criteria is reached. In `FSA`'s view of an alignment as a POSET (DAG), aligning single columns during sequence annealing is equivalent to adding relations (adding edges), maintaining alignment consistency is equivalent to ensuring that the partial order is valid (ensuring that the graph is acyclic) and producing a "global" alignment is equivalent to choosing a linear extension (choosing a topological order).

This graph-based approach to sequence alignment, which is used by programs including `DIALIGN` [13], `POA` [10], `AMAP` and `FSA`, was given an early graph-based formalization in [14]. The resulting algorithm in [14] is qualitatively similar to the sequence annealing approach which `AMAP` and `FSA` use. `DIALIGN`'s approach [13], wherein homologous segments of sequences, rather than individual characters, are aligned, uses results from this formalization [15].

As a greedy algorithm, sequence annealing first aligns the columns which will give the largest (incremental) increase in the expected accuracy of the alignment (strictly speaking, the first columns aligned have maximum weight; see below). `FSA` therefore internally creates a series of alignments with increasing expected sensitivity and decreasing expected positive predictive value or precision. The final alignment, which has the highest expected accuracy, is the one most relevant in the majority of applications, but the user can view all intermediate alignments produced by the sequence annealing process with the GUI.

In the current implementation in `FSA`, sequence annealing is a steepest-ascent hill-climbing algorithm for maximizing the expected accuracy criterion, and as such will find a local, rather than global, optimum of the objective function. Most common variants of the multiple alignment problem have been proved to be NP-hard [16], strongly suggesting that the expected accuracy approach described here is NP-hard as well. However, our experiments with simulated annealing strategies, which have given minimal performance increases, suggest that the expected-accuracy alignment landscape is relatively smooth at the large scale, thereby permitting a greedy approach to be very effective. We have additionally observed that the majority of the steps taken by the greedy algorithm (the first 80-90% on typical datasets) involve aligning characters whose homology is completely certain under `FSA`'s model, further suggest-

ing that a greedy strategy is effective on typical accuracy landscapes. Sequence annealing (the greedy algorithm) followed by iterative refinement appears to be the best strategy to use.

The order in which columns are aligned is given by a weighting function on the posterior probabilities of alignment. For two columns, each containing a single character ($x_i$ and $y_j$), the weighting function is just

$$2 \cdot \mathbb{P}(x_i \sim y_j | X, Y) \Big/ (\mathbb{P}(x_i \sim - | X, Y) + \mathbb{P}(- \sim y_j | X, Y))$$

(this is the "tgf" weighting function described in [11]). If the two columns each contain one or more characters, then the numerator ("match probabilities") and denominator ("gap probabilities") become sums over the characters pairs which are newly-aligned after aligning the two columns (see Theorem 7.2).

Sequence annealing begins by constructing a heap of all possible alignments of (edges between) single characters using this weighting function. At each step of the algorithm, a single candidate edge is popped from the heap. If the columns joined by the edge have been modified since the weight was last calculated, then the weight is re-calculated as specified in Theorem 7.2. If the new weight is no longer greater than the weight of the next edge on the heap (if the heap ordering is incorrect), then the edge is re-inserted into the heap and the next edge is popped. If the edge does have the highest weight (if the heap ordering is correct), then the edge is added to the alignment if it is not inconsistent with edges added previously. Treating a multiple alignment as a DAG, sequence annealing quickly performs this consistency-checking of candidate edges using the Pearce-Kelly algorithm [17] for online topological ordering of directed graphs. While we have phrased the algorithm in the edge-insertion framework, the implementation uses a node-contraction framework, wherein two nodes are merged when they are connected by an accepted edge.

The sequence annealing algorithm described above and introduced in `AMAP` has unfavorable computational complexity of $O(N^4)$ for aligning $N$ sequences. Consider the simple case of building a complete alignment of $N$ sequences, for which there will be $O(N^2)$ edges if we perform an all-pairs comparison. Because a complete alignment of $N$ sequences requires only $O(N)$ edges to assemble, the majority of the $O(N^2)$ candidate edges must be eventually rejected due to consistency constraints. Most of these will not be examined (popped from the heap) until the alignment is largely complete. This introduces an enor-

mous computational cost: re-weighting a candidate edge between two completely-assembled columns costs $O(N^2)$ due to the sum-of-pairs operation required, and so in the worst case, re-weighting of these candidate edges will therefore cost $O(N^2)$ for each edge. Because the number of inconsistent edges grows as $O(N^2)$, whereas the number of consistent edges grows only as $O(N)$, the sequence annealing algorithm of [11] has a worse-case complexity of $O(N^4)$, making it impractical for many sequences.

The sequence annealing algorithm therefore has two costly parts, consistency-checking with the Pearce-Kelly algorithm and edge re-weighting. While the analysis above may suggest that it is best to always perform consistency-checking before edge re-weighting, this is only clearly true once the alignment is largely assembled. There are two phases in the alignment construction, an initial phase, in which most candidate edges are accepted and the basic structure of the alignment is assembled, and a completion phase, in which most candidate edges are rejected and the structure of the alignment changes little. In the initial phase it is best to perform re-weighting first, since re-weighting is cheap on a sparse graph and most edges will pass the consistency checks; in the completion phase it is best to perform consistency-checking first, since re-weighting is expensive and most edges will fail consistency checks.

Finding an optimal solution to this trade-off is an open problem. `FSA` uses a hybrid approach, wherein it performs several fast partial consistency checks first, such as checking that candidate edges do not map to a single column of the current alignment, re-weights edges which pass these checks, and only then performs a "full" consistency check with a search of the DAG. `FSA` avoids performing repeated searches between identical columns by maintaining lookup tables of consistent and inconsistent edges which are iteratively constructed as graph searches are performed for consistency-checking of new candidate edges. It additionally amortizes re-weighting calculations to minimize their cost and keeps track of re-weighted candidate edges to avoid the cost of re-weighting "duplicate" candidate edges which join columns of the current alignment for which we have already seen and re-weighted a candidate edge. The resulting new sequence annealing algorithm avoids the bad scaling described above, giving it an approximate complexity of $O(N^2)$ for an all-pairs approach to typical problems, with the true complexity depending on the structure of the graph. The scaling with the number of sequences is further reduced to approximately $O(N \cdot \log N)$ in --fast mode.

By Theorem 7.2, the sequence annealing algorithm is a true steepest ascent algorithm in the weighting function [11].

**Theorem 7.2** *The weight of a candidate edge $w\,(col_1, col_2)$ between columns can only decrease as more characters are aligned to the columns. Sequence annealing is therefore a true steepest-ascent algorithm in the weighting function given by*

$$w\,(col_1, col_2) = 2 \cdot \sum_{x_i:\, X \in col_1} \sum_{y_j:\, Y \in col_2} \mathbb{P}(x_i \sim y_j | X, Y) \Bigg/ \left( \sum_{x_i:\, X \in col_1} \sum_{y_j:\, Y \in col_2} \left[ \mathbb{P}(x_i \sim - | X, Y) + \mathbb{P}(- \sim y_j | X, Y) \right] \right)$$

*between two columns $col_1, col_2$. This follows from this fact:*
*If $l_1, \ldots, l_k$ and $m_1, \ldots, m_k$ are positive numbers, then*

$$\max_k \frac{l_k}{m_k} \geq \frac{\sum_k l_k}{\sum_k m_k}.$$

*(This theorem and its proof are from [11].)*

**Proof** Let $C = \frac{\sum_k l_k}{\sum_k m_k}$ and assume the contrary. Then we have

$$\max_k \frac{l_k}{m_k} < C\,,$$

from which it follows that $l_k < C \cdot m_k \, \forall\, k$. However, then $\frac{\sum_k l_k}{\sum_k m_k} < C$, which is a contradiction. ∎

We have observed that in practice, `FSA`'s sequence annealing algorithm is approximately as fast as inference. This is supported by recent theoretical analysis: The average running time of the Pearce-Kelly algorithm on a complete random graph with $n$ nodes is $O\left(n^2 \log^2 n\right)$ [18]. If we perform distance-based alignment of $N$ sequences of length $L$, then this gives a runtime of $O\left(N^2 \cdot L^2 \cdot \log^2(N \cdot L)\right)$, so in the "worst average case" for sequence annealing in which we have a near-complete graph (sequence graphs are generically sparse), sequence annealing costs only logarithmically more than inference. `FSA`'s

alignment speedup techniques can further reduce this. While this average-case analysis does not take into account the cost of re-weighting candidate edges, as described above, `FSA`'s revised annealing algorithm reduces the cost of re-weighting to approximately $O(N^2)$, allowing the bound to hold.

**Theoretical justification of distance-based alignment.** Our distance-based approach to the multiple alignment problem can be viewed as an approximation to more complex models of multiple alignments. Analogously to the case of Neighbor-Joining, which uses only pairwise comparisons to approximate full models of likelihoods on trees, distance-based alignment uses only pairwise inference of alignment probabilities to approximate complete models of sequences evolving on trees.

Consider finding the optimal multiple alignment of sequences $X_1, ..., X_N$ related by a phylogenetic tree $\mathcal{T}$, so that our statistical model defines probabilities $\mathbb{P}\left(\mathcal{A}|X_1, ..., X_N, \mathcal{T}\right)$ over multiple alignments $\mathcal{A}$ given a tree $\mathcal{T}$. The generalization of Equation 2 to this case is straightforward:

$$\mathcal{A}_{optimal} = \operatorname*{argmin}_{\mathcal{A}^*} \mathbb{E}\left[d\left(\mathcal{A}^*, \mathcal{A}|\mathcal{T}\right)\right]_{\mathbb{P}(\mathcal{A}|X_1,...,X_N,\mathcal{T})} .$$

We can rewrite the objective function as

$$\mathbb{E}\left[d\left(\mathcal{A}^*, \mathcal{A}|\,\mathcal{T}\right)\right]_{\mathbb{P}(\mathcal{A}|X_1,...,X_N,\mathcal{T})} = \sum_{\mathcal{A}} \mathbb{P}\left(\mathcal{A}|X_1, ...X_N, \mathcal{T}\right) d\left(\mathcal{A}^*, \mathcal{A}|\mathcal{T}\right) \tag{5}$$

$$= \frac{1}{\binom{N}{2}} \sum_{i,j} \sum_{\mathcal{A}_{ij}} \sum_{\mathcal{A}|\mathcal{A}_{ij}} \mathbb{P}\left(\mathcal{A}|X_1, ..., X_N, \mathcal{T}\right) d\left(\mathcal{A}^*, \mathcal{A}|\mathcal{T}\right) , \tag{6}$$

where in the last form we have introduced a sum over pairwise alignments $\mathcal{A}_{ij}$ of sequences $X_i$ and $X_j$ and $\mathcal{A}|\mathcal{A}_{ij}$ refers to a multiple alignment $\mathcal{A}$ constrained to contain the pairwise alignment $\mathcal{A}_{ij}$. Equation 6 is presented to show that it is meaningful to think of taking a sum-of-pairs over sequences even within the framework of a full model of sequences on trees.

Motivated by Equation 5, we derive a distance-based alignment method from this explicit model of alignments on a tree by making two restrictions. We define the distance $d\left(\mathcal{A}^*, \mathcal{A}|\mathcal{T}\right)$ between two

multiple alignments of sequences related by a tree $\mathcal{T}$ as a weighted sum of pairwise distances,

$$d\left(\mathcal{A}^{*}, \mathcal{A}|\mathcal{T}\right) = \sum_{i,j} w_{ij}(\mathcal{T})\, d\left(\mathcal{A}^{*}_{ij}, \mathcal{A}_{ij}\right)\ , \tag{7}$$

and use a pairwise approximation to the full probabilistic model,

$$\sum_{\mathcal{A}|\mathcal{A}_{ij}} \mathbb{P}\left(\mathcal{A}|X_1, ..., X_N, \mathcal{T}\right) = \mathbb{P}\left(\mathcal{A}_{ij}|X_i, X_j\right)\ . \tag{8}$$

The first restriction, on distances between multiple alignments, is related to the approximations made by distance-based phylogenetic reconstruction methods such as Neighbor-Joining [19] and BIONJ [20]. In this paper we use weights $w_{ij}(\mathcal{T}) = 1 \forall\, i, j, \mathcal{T}$, which corresponds to a phylogeny-free approach to alignment. In principle these weights can be adjusted according to the known phylogeny using the approach of Altschul et al. [21]. The second restriction, on computation of the likelihood function, is well-studied: The Pair HMM used by `FSA` is an approximation (in a precise sense; see, e.g., [22]) of pairwise stochastic models of substitutions and indels such as the Thorne-Kishino-Felsenstein '91 model [23] and its extensions. Substituting Equations 7 and 8 into Equation 5, we obtain the sum-of-pairs objective function use by `FSA` (Equation 2),

$$\mathbb{E}\left[d\left(\mathcal{A}^{*}, \mathcal{A}|\,\mathcal{T}\right)\right]_{\mathbb{P}(\mathcal{A}|X_1,...,X_N,\mathcal{T})} = \sum_{i,j} w_{ij}(\mathcal{T}) \sum_{\mathcal{A}_{ij}} d\left(\mathcal{A}^{*}_{ij}, \mathcal{A}_{ij}\right) \mathbb{P}\left(\mathcal{A}_{ij}|X_i, X_j\right)\ . \tag{9}$$

We do not have theoretical results on the degree of approximation involved in Equations 7 and 8, and so at present these approximations remain well-motivated heuristics. However, much as Neighbor-Joining was long seen as a murky heuristic before being revealed as a greedy optimization of a principled objective function [24,25], we believe that our distance-based method should yield precise approximation bounds via a more detailed understanding of possible metrics on alignments of sequences related by a tree as well as the annealing algorithm itself.

We note that the presentation above can easily be further generalized to include, for example, sequences which have undergone recombination.

# References


1. Kurtz S, Phillippy A, Delcher A, Smoot M, Shumway M, et al. (2004) Versatile and open software for comparing large genomes. Genome Biol 5:R12.

2. Slater G, Birney E (2005) Automated generation of heuristics for biological sequence comparison. BMC Bioinformatics 6:31.

3. URL `http://www.postgresql.org/`.

4. Condor. URL `http://www.cs.wisc.edu/condor/`.

5. Jukes TH, Cantor C (1969) Evolution of protein molecules. In: Mammalian Protein Metabolism, New York: Academic Press. pp. 21–132.

6. Tamura K, Nei M (1993) Estimation of the number of nucleotide substitutions in the control region of mitochondrial DNA in humans and chimpanzees. Mol Biol Evol 10:512–526.

7. Dewey CN (2006) Whole-Genome Alignments and Polytopes for Comparative Genomics. Ph.D. thesis, EECS Department, University of California, Berkeley. URL `http://www.eecs.berkeley.edu/Pubs/TechRpts/2006/EECS-2006-104.html`.

8. Holmes I URL `http://biowiki.org/DART`.

9. Morgenstern B, Stoye J, Dress A (1999) Consistent equivalence relations: a set-theoretical framework for multiple sequence alignment. Technical report, University of Bielefeld, FSPM.

10. Lee C, Grasso C, Sharlow M (2002) Multiple sequence alignment using partial order graphs. Bioinformatics 18:452–464.

11. Schwartz AS, Pachter L (2007) Multiple alignment by sequence annealing. Bioinformatics 23:e24–9.

12. Eriksson N, Pachter L, Mitsuya Y, Rhee S, Wang C, et al. (2008) Viral population estimation using pyrosequencing. PLoS Comput Biol 4:e1000074.

13. Morgenstern B, Dress A, Werner T (1996) Multiple DNA and protein sequence alignment based on segment-to-segment comparison. Proceedings of the National Academy of Sciences of the USA 93:12098–12103.

14. Abdeddaïm S (1997) On incremental computation of transitive closure and greedy alignment. In: CPM. pp. 167–179.

15. Abdeddaïm S, Morgenstern B (2001) Speeding up the dialign multiple alignment program by using the 'greedy alignment of biological sequences library' (gabios-lib). In: JOBIM '00: Selected papers from the First International Conference on Computational Biology, Biology, Informatics, and Mathematics. London, UK: Springer-Verlag, pp. 1–11.

16. Elias I (2006) Settling the intractability of multiple alignment. J Comput Biol 13:1323–1339.

17. Pearce DJ, Kelly PHJ (2007) A dynamic topological sort algorithm for directed acyclic graphs. ACM Journal of Experimental Algorithmics 11:1.7. doi: http://doi.acm.org/10.1145/1187436.1210590.

18. Ajwani D, Friedrich T (2007) Average-case analysis of online topological ordering. In: Tokuyama T, editor, Proceedings of the 18th International Symposium on Algorithms and Computation, (ISAAC 2007). Springer, volume 4835 of *Lecture Notes in Computer Science*, pp. 464–475.

19. Saitou N, Nei M (1987) The neighbor-joining method: a new method for reconstructing phylogenetic trees. Mol Biol Evol 4:406–425.

20. Gascuel O (1997) BIONJ: an improved version of the NJ algorithm based on a simple model of sequence data. Mol Biol Evol 14:685–695.

21. Altschul S, Carroll R, Lipman D (1989) Weights for data related by a tree. J Mol Biol 207:647–653.

22. Knudsen B, Miyamoto M (2003) Sequence alignments and pair hidden Markov models using evolutionary history. J Mol Biol 333:453–460.

23. Thorne JL, Kishino H, Felsenstein J (1991) An evolutionary model for maximum likelihood alignment of DNA sequences. Journal of Molecular Evolution 33:114–124.

24. Gascuel O, Steel M (2006) Neighbor-joining revealed. Mol Biol Evol 23:1997–2000.

25. Mihaescu R, Levy D, Pachter L (2007) Why neighbor-joining works. Algorithmica doi:10.1007/s00453-007-9116-4. URL `http://www.springerlink.com/content/w3206717365g8m01`.
